# Evaluation of the Economic, Environmental, and Social Impact of the Valorization of Grape Pomace from the Wine Industry

**DOI:** 10.1021/acssuschemeng.3c03615

**Published:** 2023-09-01

**Authors:** Manuel Taifouris, Mahmoud El-Halwagi, Mariano Martin

**Affiliations:** † Department of Chemical Engineering, University of Salamanca, Plza. Caídos 1-5, 37008 Salamanca, Spain; ‡ Department of Chemical Engineering, Texas A&M, 3122 TAMU, 100 Spence St., College Station, Texas 77843A, United States

**Keywords:** grape pomace, economic, environmental, social impacts, pyrolysis, anaerobic digestion

## Abstract

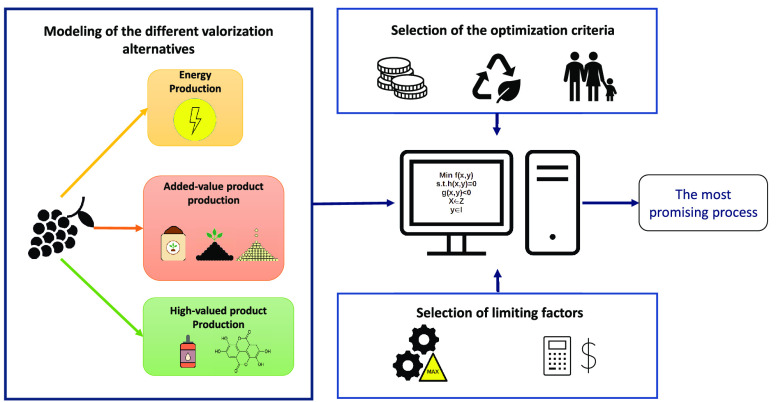

The increase in the world population has led to intensive food production systems that are generating increasing amounts of solid waste. In this work, the valorization of the most important waste generated during wine production, grape pomace, is evaluated. Eight processes are proposed to approach different types of valorization (production of energy and value-added products), from economic, environmental, and social points of view. The best process depends on the budget available, the production capacity, and the weight of each impact produced by the factory (economic, environmental, or social). For small (less than 0.1 kg/s) or very large (greater than 10 kg/s) capacities, the production of high-value-added products outperforms the other processes in all three impacts and in profitability. For intermediate capacities, combustion and gasification stand out as having the highest greenhouse emissions and intermediate economic benefits. Anaerobic digestion is remarkable for its low greenhouse gas emissions, while tannin production is the best-balanced process from both economic and environmental points of view. Pyrolysis is the worst process of all three impacts.

## Abstract 2

Multiscale methodology for the optimal valorization of wine production wastes.

## Introduction

The growth of the world population has resulted in the intensification of food production processes, which results in an increase in the amount of organic solid waste produced annually. This situation leads to an increased risk of nutrient pollution as long as they are not treated properly.^1^ This, together with greater environmental awareness on the part of governments, which has resulted in environmental policies,^2^ has pushed companies to change their production systems. The design of these new processes takes into account the concepts of circular economy and zero-emission philosophy.^3^ One of the largest contributors to solid waste generation in the food industry is wine production,^4^ especially in Italy, France, Spain,^5^ and California.^6^ During the wine production process, up to 200 kg of solid waste is generated per 750 L of wine produced. Of this solid waste, 60% consists of a mixture of grape skins and seed, representing the grape stalks, wastewater, and wine lees the rest. This waste is known as grape pomace.^7^ Grape pomace is often deposited in large aeration tanks,^4^ which does not only cause a massive loss of value but can also cause nutrient pollution due to its high concentration of organic matter.

There is a wide variety of techno-economic studies that advocate the possibility of obtaining economic and environmental benefits by using this residue as a source of value-added products and energy. Grape pomace is an important source of polyphenols and essential oils, which are antioxidant, antimicrobial, anti-inflammatory, and anticarcinogenic, and can be used as food additives or pharmaceuticals.^7^ In addition to these products, chemical, physical, and biological processes can be used to produce fertilizers,^8^ biochar,^9^ tannins,^10^ and biofuels such as biodiesel^11^ and bioethanol.^12^ The composting of grape pomace allows this residue to be used to improve soil properties or as animal feed.^13,14^ Finally, grape pomace can be used to produce power directly through thermal processes such as combustion, gasification, or pyrolysis.^15^

However, these studies usually cover a limited set of processes applied to very specific cases (production capacity or grape pomace composition), which are studied separately. The economies of scale associated with the production capacity of the treatment plant, together with a production yield dependent on the composition of the waste, means that these processes cannot be directly compared. Therefore, it is difficult to select the best option for different production capacities, physicochemical properties of the residues, and capital available for investment. On the one hand, it is not possible to evaluate the synergies that may exist between processes, such as secondary waste valorization, energy, and water integration or shared supply chains. On the other hand, many of these studies only focus on the economic dimension, leaving aside the environmental and social impacts of each process. To the best of the authors’ knowledge, there is no work that simultaneously analyzes all of the types of grape pomace valorization (energy valorization and production of value-added products), evaluating the economic, environmental, and social impact of each process, for different production capacities and budgets, under the same framework and estimation methods.

The concept of integrated biorefineries represents the optimal approach to treat organic residues^16^ due to the complexity of their compositions. These biorefineries make it possible to obtain a set of value-added products by integrating a series of chemical and physical processes at the same time, in series or parallel lines, in a single facility. This reduces the waste generation by taking advantage of synergies, such as energy and water integration and secondary waste treatment, among others. Besides, the profitability of the process is higher due to the generation of a wider range of products.^17^ Although it has been widely studied in recent years,^16,18^ this type of biorefinery requires an investment capital that may be too high for small wineries.^7^ It is therefore very important to analyze both simple and complex processes to address wine industries with different production capacities and available capital.

Therefore, in this work, a framework, that contains 8 different processes of grape pomace valorization is developed to analyze the more promising technology to obtain value from different points of view (economic, environmental, and social). Between the processes considered, there are two devoted to produce power (combustion and gasification); four to produce fertilizers (anaerobic digestion), biochar (pyrolysis), tannins, and essential oils (extraction-filtration system); and a last process to obtain polyphenols (extraction-purification system). Each process is modeled using first principles such as mass and energy balances, thermodynamic equilibrium, empirical correlations, and performances.^19^ This allows determining which is the best process for different capacities, for different budgets, and from different points of view (economic, environmental, and social). Furthermore, it analyzes possible combinations of processes to reduce the environmental impact and improve both the economic and social impacts. The paper is structured as follows. In the Framework Development section, we explain how the different processes have been modeled and how the economic, environmental, and social evaluation has been carried out. The Results section presents the case study used to evaluate the model designed in the previous section and shows the results of the analysis. Finally, the Conclusions section presents the most significant conclusions on the results of the research.

## Framework Development

### Estimation of the Production and Composition of the Grape Pomace

Following the work of Rodrigues et al.,^20^ it is estimated that 0.16 kg of grape pomace is produced per liter of wine. Therefore, grape pomace production is estimated from wine production, following public information about winery sales. The estimation of the composition of grape pomace is more complicated because it can change depending on the reference consulted since it is a waste that is strongly dependent on the type of grape and wine production. Therefore, it is essential that the mathematical model takes into account the grape composition in order to estimate the economic, environmental, and social impacts of the treatment processes. In order to calibrate the models presented in this section, a particular grape pomace composition estimated from different references is used and shown in Table S.3 in the Supporting Information. In spite of the fact that this study focuses on the valorization of grape pomace, some of the processes, such as anaerobic digestion, gasification, or extraction, can be used to treat the rest of the waste generated during winemaking, such as lees and wastewater.

### Processes Analysis and Design

Due to the large number of possible products and processes to obtain value from this type of waste, a prescreening was necessary to reduce the number of processes considered to eight. This prescreening consisted of analyzing the economic, environmental, and social potential, based on very simple models (empirical yields, stoichiometric balances, interpolation of experimental data, among other techniques).

The processes shown in this Section are modeled following first principles, such as mass balances, energy balances, and thermodynamic equilibria as well as empirical correlations or yields, based on information from different works used as references. Since there is a model for each process, the optimization framework consists of eight mathematical models. Each model is optimized to maximize profit, although the environmental and social impacts are also evaluated, following the procedures described in the Economic, Environmental, and Social Impact Estimation of Each Process section. The waste treatment line is designed as an independent factory with its own workforce. This assumption is taken into account for the economic estimation of each of the processes. Although the wine production process is seasonal (from August to November), the waste treatment process, as well as the wine production process, is continuous.

The processes are divided into three groups. Combustion and gasification are aimed at producing energy from grape pomace, while the rest of the processes are used to obtain chemical products, which can be classified into valued and high-value products, depending on their market value. On the one hand, anaerobic digestion, pyrolysis, and an extraction-filtration system are used to produce fertilizer, biochar, and tannins, which constitute the group of valued products. On the other hand, an extraction-purification system is used to obtain polyphenols and essential oils, the products considered as high-value products in this work.

#### Energy Production

**Figure 1:**
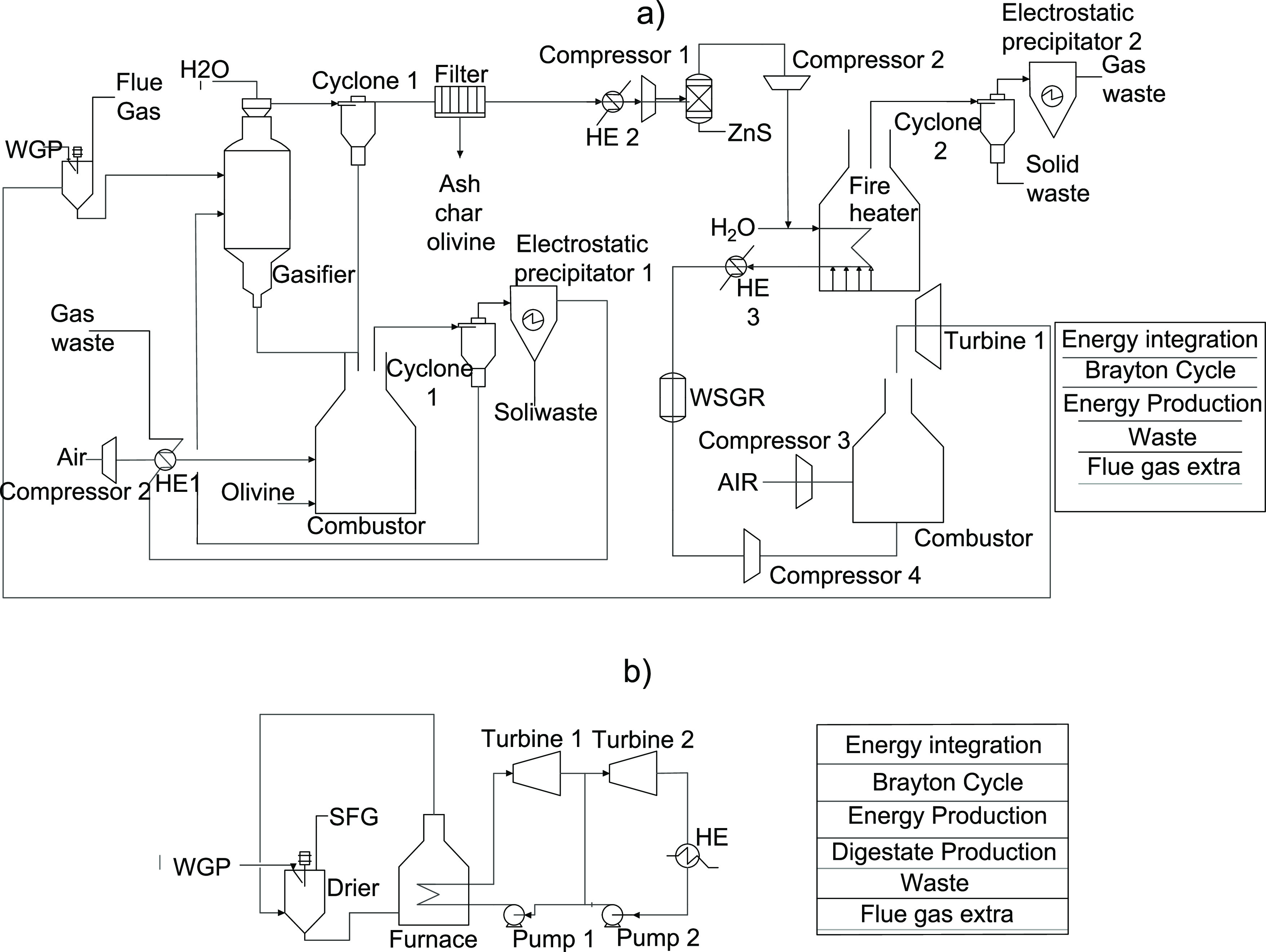
Schematic diagrams of the processes that produce power (a: gasification; b: combustion).

There are three processes that use grape pomace to obtain energy: combustion, gasification, and pyrolysis. However, in this section, only the first two are considered for obtaining energy since pyrolysis can be used to produce biochar, which is considered an added-value product. The schematic diagrams for combustion and gasification can be seen in Figure 1.

First, it is necessary to dry the raw material to 10% moisture content for both processes. Although natural drying (storing the raw material until its water composition is reduced to 10% by contact with atmospheric air) could have been considered, this involves a number of environmental impacts related to nutrient pollution,^1^ which it was preferable to avoid. Therefore, spent flue gas from the Rankine cycle (in combustion) and the Brayton cycle (in gasification) is used to dry the wet grape pomace. For the modeling of the drying process, it is necessary to estimate the specific heat of grape pomace. For this purpose, the composition of grape pomace (see Table F.3 in the Supporting Information) and the empirical correlation shown in the work of Sahin and Sumnu^21^ are utilized. The concept of specific humidity and Antoine’s law are added to the model to avoid gas saturation.

The combustion process directly utilizes the dried grape pomace to produce energy by means of a furnace. To model the energy and mass balances of the combustion process, stoichiometric ratios and the empirical formula for grape pomace (CH_1.3626_N_0.033_O_0.4766_) are used. This formula can be estimated by using the ultimate analysis of the grape pomace.^22^ An excess of 150% air is used to avoid a temperature too high in the furnace. By designing the furnace, it is possible to adjust the heat used to produce steam and the heat absorbed by the flue gas. This ratio is adjusted to produce enough flue gas to dry the raw material, while the remaining energy is used to produce power through the steam generated. The Rankine Cycle is modeled following the work of De la Fuente and Martin.^23^

Regarding the gasification process, the work of Sánchez et al.^24^ is followed. This process consists of grape pomace gasification, syngas upgrading, and a Brayton cycle. From an economic point of view, the best configuration for the gasification of lignocellulosic residues is indirect gasification.^24^ In this type of system, the heat requirement for the gasification stage is supplied by the combustion of the char in a combustor. The heat is transferred between the combustor and the gasifier through a heat transfer medium (olivine), and the char is generated in the gasification process. Therefore, this process is autothermal. The energy and mass balances in the combustion system are performed considering the total oxidation of all compounds except nitrogen in the air. A specific heat of combustion for char of 25,000 kJ/kg is taken.^25^ Regarding the mass and energy balances of the gasification, the composition of the outside gas is estimated using the temperature and the correlations of Phillips et al.^26^ The gasification is carried out with a pressure of 1.6 bar and ratios of 0.4 kg of steam and 27 kg of olivine per kilogram of grape pomace. However, it is expected that most of it will be reused (more than 99%) according to the results consulted in the literature.^26^

The solid residues (mainly ash and olivine) are captured through a series of cyclones (99% separation efficiency) and an electrical precipitator (99.99% separation efficiency). The ZnO bed is used to separate 100% hydrogen sulfide through the reaction shown in eq 1.
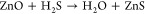
1

Subsequently, the gas is upgraded by steam reforming, removing the hydrocarbons present in the stream. Steam reforming is modeled considering that all hydrocarbons, except methane, are completely transformed into CO and H_2_ (eq 2), while the amount of CH_4_ is modeled from the thermodynamic equilibrium^27^ and stoichiometry ratios (eq 3 and 4).
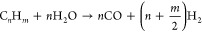
2
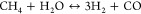
3
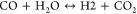
4

This process is considered adiabatic. Next, the H_2_/CO ratio must be adjusted to optimize the combustion process and the Brayton cycle. This process is also considered adiabatic and is modeled using thermodynamic equilibrium and eqs 3 and 4. Finally, a PSA system is used to remove NH_3_ and H_2_O. Due to the selectivity of the adsorbent used in the PSA tower(zeolites), CO_2_ is also adsorbed, reducing its concentration to 2%. The PSA tower is modeled using empirical performances following the literature.^28^ A Brayton cycle is used to produce energy because syngas is a gaseous fuel. Since the exhaust gases from the Brayton cycle are used to dry the feedstock, the use of a combined cycle is discarded. In this way, less power is produced, but it is not necessary to use an external heat source to dry the grape pomace.

Note that while the thermal processes look alike, the final products present several differences resulting in high CAPEX in the case of producing syngas with the proper composition and free of contaminants compared to the solid products of the pyrolysis or the direct combustion of the waste. A more detailed explanation of each process is provided in the Supporting Information.

#### Added-Value Product Production

**Figure 2:**
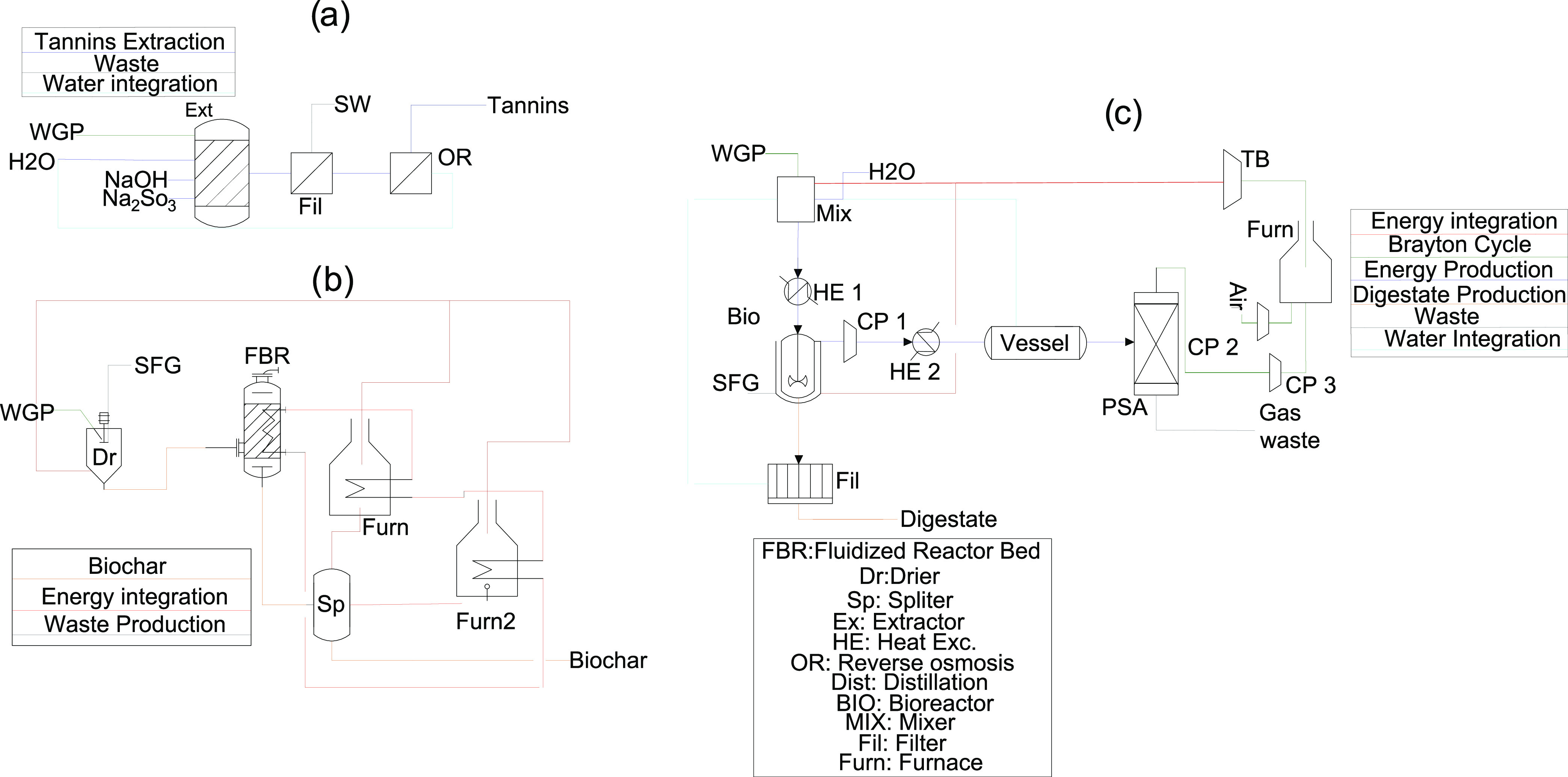
Schematic diagrams of processes that produce added-value products (a: tannins, b: biochar, c: fertilizer).

Among the possible products that can be obtained from grape pomace, up to 3 products are considered as valued in this work. These products are biochar, fertilizer, and tannins. Each product has a different production process, which are shown in Figure 2.

An extraction-filtration process is used to obtain tannins from grape pomace. In this case, the empirical results of the work of Ping et al.^10^ are used to estimate the mass and energy balances of this process. For the extraction process, NaOH (2.5% of the dry grape pomace), water (8:1 with respect to the solid phase), and Na_2_SO_3_ (2.5% of the dry grape pomace) are required. The optimum operating conditions for extraction are a temperature of 100 °C and a residence time of 120 min. A filter separates the solid residue from the liquid stream, in which the dissolved tannins are found, with a ratio of 4.38 kg of liquid for each kg of solid. The liquid stream is subjected to reverse osmosis. The concentration factor of reverse osmosis for this type of product (i.e., tannins) is of the order of 7.5.^29^ Therefore, this empirical value is used to determine the maximum amount of water that can be removed from the stream, that is, this step reduces the water content of the stream down to 13.4%. This way, much of the water used in the extraction process can be recovered. Finally, the tannins are dried in contact with air to their final moisture content (9.37%) and stored. According to this work, it is possible to produce up to 0.05 g of tannins per gram of dry grape pomace, which can be sold directly. However, a significant amount of solid residue is generated in the process (0.65 kg per kilogram of dried grape pomace). This residue has a composition very similar to that of grape pomace since most of the compounds (cellulose, hemicellulose, proteins, and fats) are not soluble in this solvent, and therefore, this residue can be used to produce fertilizer and biogas through an anaerobic digestion process, increasing the profitability of the process and reducing the environmental impact. The main electrical energy consumption of the process corresponds to the pumps used to reach the operating conditions of the reverse osmosis equipment, that is, 20 bar. The consumption of these pumps is estimated through an energy balance assuming an efficiency of 0.47.^30^

Fertilizer is produced by the anaerobic digestion of grape pomace. The work of Taifouris et al.^31^ is used to model the mass and energy balances of this process. This model uses the amount of carbohydrates (C_6_H_10_O_5_), lipids (C_57_H_104_O_6_), and proteins (CH_2.03_O_0.6_N_0.3_S_0.001_) to estimate the composition of biogas (CH_4_, CO_2_, NH_3_, and H_2_O) using empirical biodegradability yields and stoichiometric ratios (eqs 5–7). C_5_H_7_NO_2_ is the empirical formula of the cell mass.
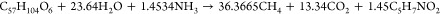
5
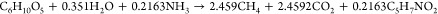
6
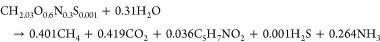
7Regarding the digestate composition, it is estimated using total solids, volatile solids, total nitrogen, organic nitrogen, and the potassium and phosphorus composition of the grape pomace. The process starts with a mixture of grape pomace with water up to a solid concentration of 10%. This mixture is heated to mesophilic conditions (37 °C) and introduced into the reactor, where it remains for 21 days.^32^ The energy requirement of the biological reaction is often difficult to estimate from the standard enthalpy of formation of raw materials and products. However, it can be estimated from empirical results from the work of Wu et al.^33^ to be 3.4 kJ/VS_degraded_. The biogas is upgraded to produce biomethane using a cooling system and a PSA tower. The cooling system is modeled using Dalton’s and Raoult’s laws, while the PSA tower is modeled using empirical yields. The digestate is dehydrated with a centrifugal filter and stored for sale as fertilizer. The biomethane is used to produce energy through a Brayton cycle, and the spent flue gas is used to supply energy to the bioreactor. Since the exhaust gases from the Brayton cycle are used to supply heat to the anaerobic digestion process, the use of a combined cycle system is discarded to avoid having to provide heat from an external source.

Biochar is produced by the pyrolysis of grape pomace. First, it is necessary to dry the raw material to 10% moisture. The procedure for estimating the energy balance is the same as for the combustion and gasification processes. The pyrolysis temperature is set at 500 °C since the biochar obtained with these operating conditions presents the maximum nutrient contents (nitrogen, phosphorus and potassium) following the results of Ferjani et al.^9^ This process is modeled using the empirical yield to estimate the amount of gas (38% of the dry pomace), bio-oil (31% of the dry pomace) and biochar (30% of the dry pomace), as well as, their compositions.^34^ The energy requirement is also estimated using empirical yields.^35^ The bio-oil and gas are used to produce energy for pyrolysis and drying of the raw material. Using the ultimate composition of the bio-oil,^34^ it is possible to estimate the empirical formula, CH_1.33_N_0.0316_O_0.179_, and model the combustion of this product. Since the gas composition is also known, modeling the combustion only requires considering the stoichiometric ratio between feedstock and products (total oxidation of all feedstock except nitrogen in the air is considered). Both flue gases are mixed to supply energy to the pyrolysis stage and to dry the feedstock. More details on each process are provided in the Supporting Information.

#### High-Valued Product Production

**Figure 3:**
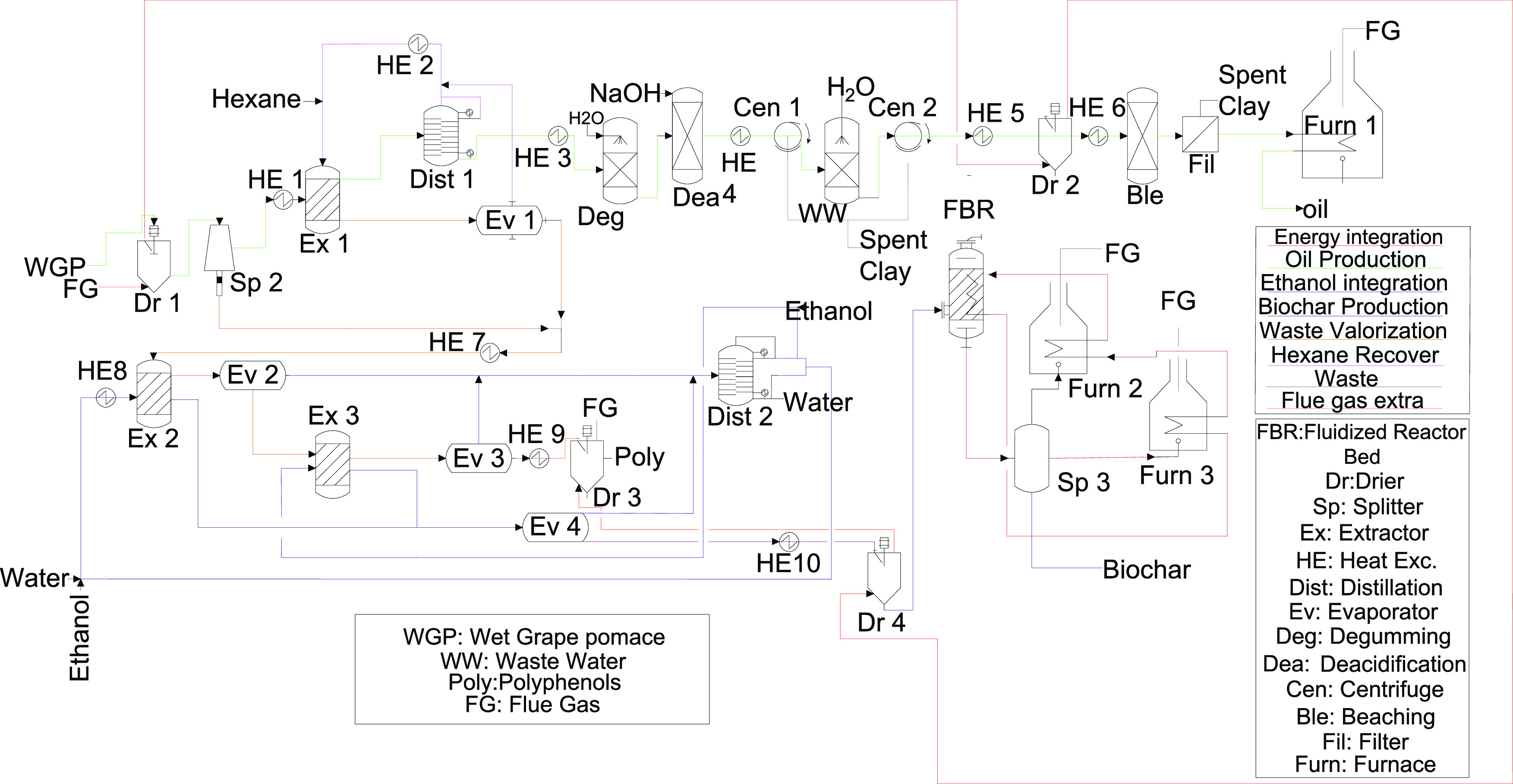
Schematic diagram of IMPS.

Through an integrated multiproduct system (IMPS), it is possible to obtain polyphenols, oil, and biochar,^7^ following the process diagram shown in Figure 3. This system consists of three combined processes, a hexane-extraction system to produce oil, an ethanol-extraction system that uses the residues of the first one to produce polyphenols, and, finally, a pyrolysis process that converts the remaining solid residues into biochar and energy. Ethanol and hexane have been used because of their production within biorefineries as well as because they are widely used in the literature for this purpose.^36,37^ Since it is a process that integrates a large number of stages, the capital investment required is expected to be high. Therefore, grape pomace can only be used to produce oil if there is not enough capital to invest in the complete process. For this reason, the oil production process is considered as a possible independent process. Because of the wide variety of equipment used in this process together with its specific application for this type of waste, the total electricity consumption (both for the integrated system and for the oil production) is estimated from the work of Jin et al.,^7^ considering a linear relationship with the grape pomace fed to the system. The electrical energy and steam required for both systems are produced through the combustion of part of the feedstock.

The seed oil is obtained by an extraction-purification process using hexane as solvent. For this purpose, the work of Jin et al.^7^ is used as a reference. To estimate the mass and energy balances, the information on the process is used as well as the design of the equipment shown in the Supporting Information of that work. First, it is necessary to dry the raw material. Therefore, a part of the grape pomace (12.28% of the dry grape pomace) is sent to a furnace to produce flue gas, which is used to reduce the amount of water down to 7.8% moisture in the grape pomace used to produce seed oil. The seeds (64%) are separated from the skins (36%) by sieving and crushing to facilitate the extraction process. The seed is fed into the extractor together with hexane (3:1 with respect to the dried grape seed). This treatment recovers 98.7% of the grape seed oil. The optimum temperature of the extraction is 60 °C. After extraction, the solvent is removed in both solid (evaporation) and liquid (distillation) phases. NaOH (0.2% of the seed oil) is used for the oil deacidification phase (60 °C), while H_2_O (30% of the seed oil) is added to remove the soapy fraction present in the oil. This stage is carried out at a temperature of 80 °C. The oil is dried (to 0.1% moisture) and clay (3% of the seed oil) is used to adsorb coloring components at a temperature of 115 °C. To model the energy balance of the drying process, the specific heat of the oil is estimated following the empirical correlation of the work of Sahin and Sumnu.^21^ Finally, a furnace is used to remove odors from the oil (230 °C). However, this process presents a major drawback, since a significant amount of solid waste is generated (0.49 kg of solid waste per kilogram of dry pomace) as well as used soap and the spent clay.

The solid residues produced during seed oil production (including the skins of the grape pomace) can be sent to a new extractor that uses an ethanol solution (40% concentration) as the solvent (5:1 with respect to the solids fed). This treatment recovers 82.8% of the polyphenols from grape pomace. The optimum temperature of the extraction is 70 °C.^7^ A decanter centrifuge is used to separate both phases in a relation of 1.75 kg of liquid per kg of solid. The solvent is recovered by means of a two-effect evaporator. For the mass and energy balances of this stage, as well as the rest of the stages focused on ethanol recovery, the feed is considered as an ethanol–water system. Since it is possible to estimate the mass balances of this equipment from the results of the work of Jin et al.,^7^ the operating temperature of the equipment can be determined from the equilibrium data of the ethanol–water system. The temperature of this equipment is 97 °C. A decanter centrifuge and a disk centrifuge are used to separate both phases in a relation of 1.31 kg of liquid per kg of solid. All polyphenol-enriched polymers are considered to be only recovered with the liquid phase. The polyphenol-enriched stream is subjected to a second extraction with ethanol (95% concentration) at a 2:1 ratio with respect to the feed. The solvent is recovered by evaporation (79 °C), and the stream with polyphenols is dried to 7% moisture. For modeling the evaporation and drying processes, it is necessary to estimate the specific heat of the polyphenols. For this purpose, the work of Erkac and Yigitarslan^38^ is used. As regards the solid phase, it is separated from the ethanol by evaporation and used as feedstock for a pyrolysis process, to obtain biochar and energy following the process described in the previous Section and with the same operating conditions. The estimation of the specific heat of the solid product is necessary to model the energy balance of the evaporation process. For this purpose, the composition of the solid is considered to be similar to that of grape pomace but without the oil fraction. All streams consisting of a mixture of ethanol and water are mixed and fed to a distillation tower to obtain ethanol, with a concentration of 95%, and water. The ethanol and water are reused in the process, reducing the economic and environmental costs of the process. More details of each process are shown in the Supporting Information.

### Economic, Environmental, and Social Impact Estimation of Each Process

In order to facilitate decision-making, the most representative index for each impact considered (economic, environmental, and social) was selected. Some indices that evaluate the economic impact of a facility are profit, NPV, or ROR.^30^ For the sake of simplicity, when comparing the different processes, the profit is used as the economic index. This index is calculated using the income from the sale of the products and the OPEX of the processes (eq 8).
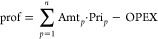
8

where Amt_*p*_ is the amount of the product “p” and Pri_*p*_ is the price of the product “p”. OPEX consists of a variable part (raw material cost and utilities) and a fixed part (maintenance, labor, laboratory costs, capital charges, among others). Product income and the variable part of the OPEX (cost of raw material and utilities) are estimated using mass and energy balances for each process, as well as updated prices, which can be consulted in Table S6 in the Supporting Information.

The fixed part of the OPEX is estimated following the procedure shown in Sinnot.^30^ Therefore, the OPEX is calculated by eq 9.
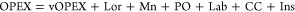
9

where vOPEX is the variable part of the OPEX, while the rest of the costs constitute the fixed part of the OPEX (fOPEX); Lor is the cost of Labor (15% of the OPEX); Mn is the maintenance (5% of the fixed capital); PO is the plant overhead (50% of the labor cost); CC is the capital charges (5% of the fixed capital); and Ins is the insurance (1% of the fixed capital). Therefore, the OPEX can also be calculated as a function of the vOPEX and fixed capital, following eq 10.
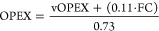
10

It is necessary to calculate the CAPEX of the factory to estimate the fixed operating cost.^30^ CAPEX is estimated by following different procedures described in the literature, depending on the process, as indicated in Table 1. For further details, refer to the Supporting Information.

**Table 1:** CAPEX Estimation of the Processes Considered

process	references
combustion	(39)
gasification	(24,39,40)
anaerobic digestion	(31,39)
pyrolysis	(15)
IMPS	(7)

Besides, the costs are updated using the CEPCI indexes.^41^ Once the cost of each piece of equipment has been calculated, the fixed capital cost is estimated following a factorial method described in the work of Sinnot.^30^

In addition, the rate of return (ROR) on investment is used to analyze the profitability of each process. It is calculated following eq 11. It is assumed that in the first two years, there is no revenue and that taxes are 30%^30^ of the annual gross profit.
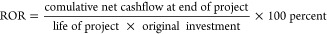
11

The most complete method for analyzing environmental impact is the life cycle assessment (LCA).^42,43^ However, the use of fully detailed LCA complicates the comparison between processes due to the ambiguity when weighing each of the possible environmental impacts they evaluate (impact on the atmosphere, soils, etc.). In addition, it is necessary to take into account that most of the environmental impact of the processes considered is due to the emission of gases into the atmosphere. For this reason, the global warming potential (GWP) is chosen as the most appropriate index for the analysis of the environmental impact of the processes. GWP is calculated by eq 12.
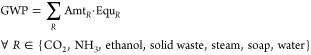
12

where Amt_*R*_ is the amount of each residue generated and Equ_*R*_ is the CO_2_ equivalent. Following eq 12, the different compounds of the gaseous wastes, as well as the solid wastes generated, are transformed into equivalent CO_2_ using the values shown in Table S7 in the Supporting Information.

Finally, with respect to social impact, several indices can be considered, such as employment generated, worker health and safety, social equity, land use and agriculture, or social acceptance and cultural aspects.^44^ However, since this is a feasibility study, where detailed engineering of each process is not carried out, it is considered to use employment generated as the most representative social impact, following other similar studies in the literature.^45^ Since labor cost (direct jobs) often represents between 10 and 20% of the operating cost^30^ and it is estimated that 7.5^46^ indirect jobs are created for each direct job, the total number of jobs created by investing in grape pomace processing can be calculated using eq 13.
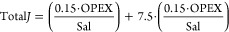
13

where Total_*J*_ is the total number of jobs created and ’Sal’ is the salary that can be estimated depending on the country where the factory is located. When direct jobs are less than 5, this equation cannot be used since at least one person per shift is needed to maintain a continuous process. In this case, the number of direct jobs is 5 and the labor cost must be assumed to be more than 15% of the operating cost.

These indexes are normalized using the min/max method (eq 14) to facilitate comparison between processes.
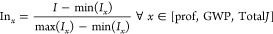
14

where *x* consists of objective variables (profit, CO_2_eq, and number of jobs), min(*I*_*x*_) is the minimum value of these variables among all of processes considered in this work, and max(*I*_*x*_) is the maximum value. The impact of each index must be analyzed individually. The higher these indices are in the case of social and economic impact, the better it will be for society and for the company. However, the higher the environmental impact index, the worse it is.

## Results

Transportation of biomass waste is difficult due to its low density and decomposition over time, which increases its transportation cost and hazardousness. Therefore, the processes considered in this work are intended to be part of the winemaking process. Moreover, in this way, it is possible to better assess the amount and composition of grape pomace, which is very important for the design and control of waste treatment. Due to the complexity of the processes presented, especially the gasification process and IMPS, a minimum treatment capacity of grape pomace is necessary for these processes to be economically profitable. After a preliminary economic study using the models described in the Processes Analysis and Design section, it is determined that the minimum capacity is 0.1 kg/s of grape pomace for at least one of the processes to be economically profitable. For those wineries with a lower production capacity, it would be necessary to evaluate other alternatives with lower CAPEX and lower OPEX, such as the composting process.

Analyzing the largest wineries in California, their production ranges from 2 million cases (9-L boxes) to 53 million cases.^47^ Therefore, these industries can generate between 18 and 477 million liters of wine per year. This is equivalent to grape pomace production between 0.1 and 2.5 kg/s (see the Estimation of the Production and Composition of the Grape Pomacesection). The production of these wineries represents almost 40% of the total wine production in California. Therefore, if a treatment line of the grape pomace is built in all of these wineries, it is not necessary to use any type of transportation to valorize almost half of the grape pomace produced in this state of the USA. Following these production capacities, 3 sizes are considered to address the best treatment process for each type of winery, which are classified as small (0.1 kg/s of GP), medium (1 kg/s of GP), and large (10 kg/s of GP). The optimization framework consists of eight different mathematical optimization models. Each mathematical model is optimized separately, and a sensitive analysis is performed to select the best option for different capacities and investments, from economic, environmental, and social points of view. If the solution to be implemented is not accepted by the winery managers, this study shows and ranks different alternatives with their economic, environmental, and social issues so that a more suitable technology can be considered

### Analysis of the Optimal Process by Type of Product

**Figure 4:**
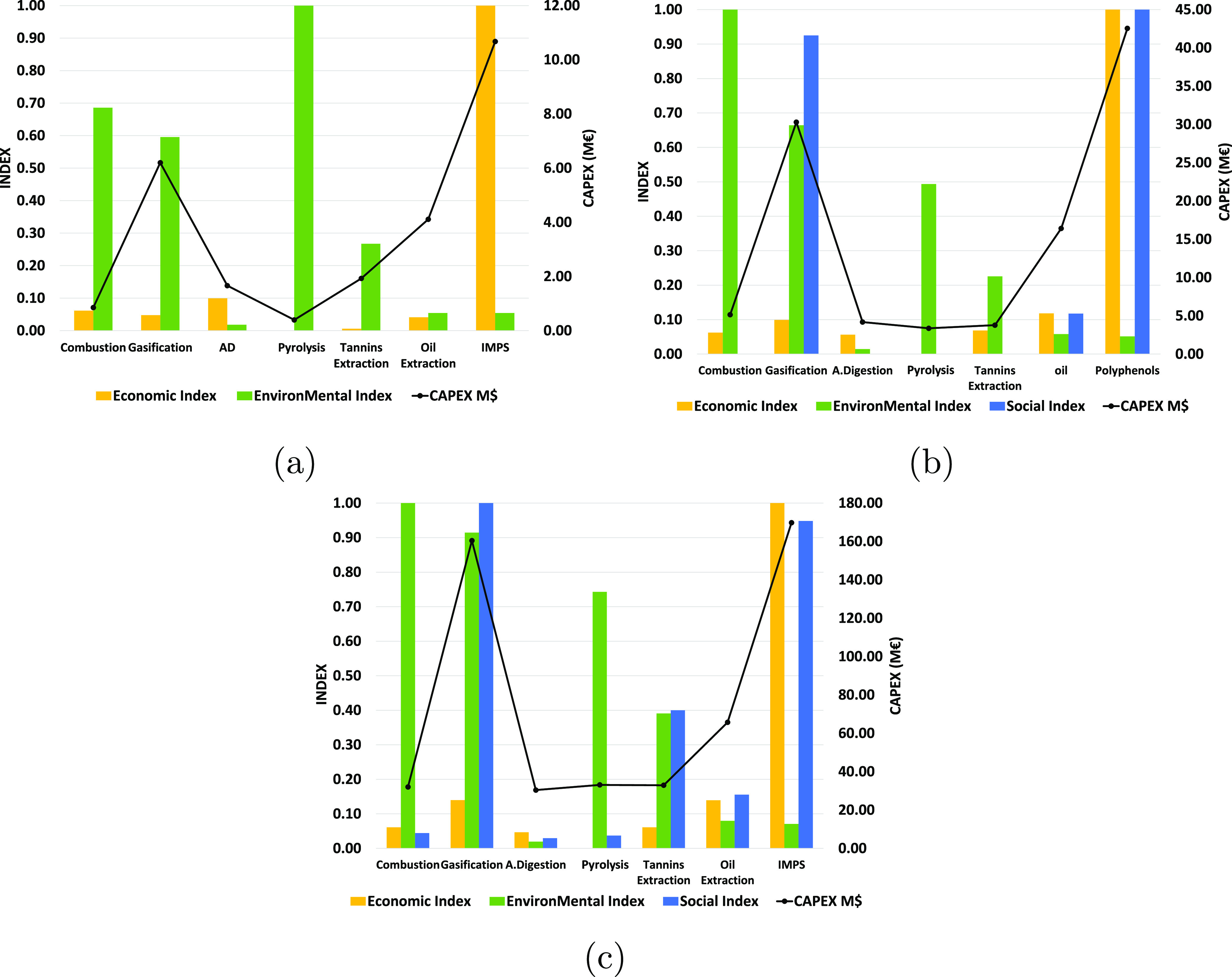
Economic, environmental, and social impact of each process for the three capacities considered (a: 0.1 kg/s, b: 1 kg/s, c: 10 kg/s).

Each of the processes described in the Processes Analysis and Design section are evaluated and optimized for the case studies described in the Results section. From the results, the economic, environmental, and social impact indices are calculated for each of the processes and are shown in Figure 4. These are used to compare each of the processes considered. The results of the material balances, as well as the investment cost (CAPEX) and the operational costs (OPEX) of each process, can be found in Tables S8 and S9 in the Supporting Information.

Between the energy production processes, that is, combustion and gasification, similar economic and social impacts are observed for the case of 0.1 kg/s of DGP. However, the difference is larger as the capacity increases. This is because gasification allows the production of up to 3 times more energy with the same amount of raw material and with a lower emission of greenhouse gases. This becomes even more evident in the last scenario considered (10 kg/s) where economies of scale allow a much higher economic and social impact in the case of gasification. However, the process is much more complex, requiring a much higher CAPEX (5 times higher, see Figure 4). This also allows for a larger social impact by generating a greater number of jobs.

With respect to the added-value products, that is, the production of fertilizer, biochar, and tannins, it can be observed that pyrolysis has the worst economic impact among all processes. Moreover, its environmental impact is also the highest among the processes oriented to producing chemical products. This is due to the need to dry the raw material, together with the low value of the biochar. Unlike pyrolysis, anaerobic digestion does not require drying of the feedstock and allows for obtaining electrical energy through biogas combustion. This has a greater economic impact than pyrolysis and tannin production for the 0.1 kg/s case. However, the economics of scale allow tannin production to have a larger economic benefit than anaerobic digestion in the 1 and 10 kg/s cases. In these cases, the best process depends on which index is given more weight, the economic or environmental impact, since tannin production has a much larger environmental impact. Although neither process requires drying of the raw material, in the case of tannin production it is necessary to burn part of the grape pomace (which produces CO_2_) to generate steam to raise the temperature of the raw materials to the conditions of the extraction process (100 °C). In addition, tannin production also has a higher social impact for the 1 and 10 kg/s scenarios given their higher CAPEX.

Finally, oil extraction and IMPS have the highest economic benefits among all processes (with the exception of gasification in the 10 kg/s scenario). The environmental impact is similar between both processes, being lower in the case of IMPS due to the treatment of the solid residues generated in the oil production process (see the High-Valued Product Production section). In addition, IMPS is also much better than oil extraction from the economic and social points of view. This is due to the high market value of polyphenols and the large capital investment required for their production, raising the OPEX and therefore the amount of money available for hiring employees. Therefore, the best process to obtain high-added-value products is the IMPS, analyzing any of the considered indexes. However, it should be noted that hexane extraction makes it difficult to use oil in the food industry due to its toxicity.

### Analysis of the Optimal Process by Invested Capital

The most promising process depends on three factors: the available capital for investment, processing capacity, and weight of each index. The necessary CAPEX for each process can be consulted in Figure 4. By analyzing the figure, it can be observed that for all capacities, there are two processes that require much higher CAPEX than the rest, gasification and IMPS. The combustion, anaerobic digestion, pyrolysis, and tannin extraction processes have very similar CAPEX to each other and much lower than gasification and IMPS. Finally, the oil extraction process has intermediate CAPEX between the two previous groups.

If there is a large amount of available capital for investment, enough to choose between the IMPS, gasification, or oil extraction processes, then the most promising process is the IMPS, from both economic and social points of view and for any capacity. Regarding environmental impact, only anaerobic digestion and tannin extraction (for the case of 1 kg/s) have an environmental impact lower than that of this process. However, the difference in economic benefit is so significant that it would be necessary to weigh the environmental impact heavily to compensate for it.

In the case that the available budget for waste treatment is insufficient to implement the IMPS or gasification process, but sufficient to select the oil extraction process, then the most promising processes are anaerobic digestion (for a capacity of 0.1 kg/s) and oil extraction (for capacities of 1 and 10 kg/s). This is mainly due to the different effects that economies of scale have on the processes. The complexity of the oil extraction process means that for small capacities the revenues from the sale of oils do not allow for profits as high as in the case of anaerobic digestion. This allows anaerobic digestion to be the most promising process for this capacity and budget limitation. However, for larger capacities, the most promising process is oil extraction since it is better than anaerobic digestion in two indices (economic and social), better than tannin extraction in two indices (economic and environmental), and better than combustion and pyrolysis in all indices.

If the budget is even more limited so that none of the previous three processes can be selected, the analysis becomes more complicated since the economic impacts of the remaining four processes (combustion, anaerobic digestion, pyrolysis, and tannin extraction) are very similar for capacities of 1 and 10 kg/s. For a capacity of 1 kg/s, fertilizer production and tannin production are balanced, while combustion is the worst in all indices. Depending on the weight assigned to the environmental impact, one or the other is chosen as the best process due to its significant difference in this index (tannin production produces 21 times more CO2eq than anaerobic digestion). Finally, in the case of 10 kg/s, the results are similar to those in the previous case.

### Determination of Optimal Investment by Production Capacity

A feasibility analysis is carried out to determine the best capital investment, if available, based on the profit and CAPEX of each of the grape pomace treatment processes. For this purpose, the ROR on investment of each process for each capacity is used. The results are shown in Figure S2 in the Supporting Information.

These results show that, for the highest production case, that is 10 kg/s, there is one process that is much more profitable than the rest, the IMPS. On the contrary, in the case of 0.1 kg/s, there are several processes that are not profitable (combustion, pyrolysis, and tannin production). In this scenario, the only promising process is the IMPS. Therefore, for both capacity (0.1 and 10 kg/s), it is recommended that sufficient investment be made to implement the IMPS, provided that it is possible to do so. However, in the intermediate capacity (1 kg/s) there are several processes with very similar ROR. On the one hand, IMPS has a ROR identical to tannin extraction, but with a much higher CAPEX (5.15 times, see Figure 4). On the other hand, anaerobic digestion has a ROR very similar to combustion but with a much lower environmental impact (11 times). In this case, it is better to opt for a smaller investment that involves less financial exposure.

## Conclusions

This paper presents an economic, environmental, and social analysis of 8 different processes for the valorization of one of the most important wastes generated during wine production, grape pomace. The processes are modeled, through mass balances, thermodynamic equilibria, empirical correlations, and performances. After analyzing the economic feasibility studies, there is a strong incentive to treat these wastes to obtain value-added products, reducing the environmental impact of the wine production process and improving the social and economic impact of the entire process. The models are applied to a case study with 3 different production capacities, 0.1, 1, and 10 kg/s.

After economic, environmental, and social analysis of each of the processes, it was found that the determination of the most promising process depends on the capital invested, the production capacity of grape pomace, and the weight of each of the indices that measure the economic, social, and environmental impact. If sufficient capital is available, the suggested process from economic and social points of view is the integrated multiproduct system, which produces polyphenols, oil, and biochar, for capacities below 0.1 kg/s and above 10 kg/s. In fact, it is the only one that is really profitable for capacities of less than 1 kg/s. However, it is necessary to highlight that the toxicity of hexane complicates the use of the extracted oil in the food industry, opening the possibility of investigating these integrated processes for different solvents, such as supercritical CO_2_ or ethanol. Only in the intermediate capacity case (1 kg/s), it may be interesting to invest in the tannin production process, if the economic and social impacts are prioritized, or in anaerobic digestion, if the environmental impact is prioritized over the other two. Energy processes are discarded because they are not competitive from an economic and environmental point of view, similar to the pyrolysis process.

It is concluded that if sufficient capital is available, the treatment capacity is higher than 0.1 kg/s, and the technology is chosen correctly, the treatment of this type of waste is not only economically profitable but also reduces the environmental impact of the wine production process, favors the circular economy of waste, and has a positive social impact, generating a large number of jobs. However, for this investment to be as efficient as possible, it is necessary to select the most suitable process according to the weight of each target, the available capital, and the production capacity, following the results shown in this research.

Although the environmental impact has been reduced by recovering the waste, there are still a number of wastes that are not fully treated. Therefore, it is the subject of future studies to reduce the footprint of these processes on the environment. These conclusions correspond to a specific residue composition that is considered constant over time for a particular winery. If there is a change in the winery, then the waste composition must be determined by adjusting the optimization framework. This framework is flexible enough to accept a wide range of compositions.

## Supporting Information Available

Mathematical models of the processes considered, additional information on the economic and environmental estimation of the processes, and the detailed results of the techno-economic analysis of each process (PDF)
