# Supplementary material for: Evaluation of the Economic, Environmental, and Social Impact of the Valorization of Grape Pomace from the Wine Industry
Source: ACS Sustain Chem Eng. 2023 Sep 1;11(37):13718–28. doi: 10.1021/acssuschemeng.3c03615 (PMC10521143; doi:10.1021/acssuschemeng.3c03615)
Supplement: Supplementary file 1 — sc3c03615_si_001.pdf [file sc3c03615_si_001.pdf]

Supporting Information
Evaluation of the economic, environmental and social impact of the valorization of grape pomace from the wine industry Supporting Information

Manuel Taifouris,† Mahmoud El-Halwagi,‡ and Mariano Martin*,†

†*Department of Chemical Engineering, University of Salamanca, Plza. Caídos 1-5, 37008 Salamanca, Spain*

‡*Department of Chemical Engineering, Texas A&M, 3122 TAMU, 100 Spence St. 77843A, College Station, USA*

E-mail: mariano.m3@usal.es

Number of pages: 38

Number of tables: 9

Number of figures: 2

Framework development

The following sections present the modeling of the proposed processes to treat the grape pomace, that is, combustion, gasification, anaerobic digestion, pyrolisis, oil extractions and polyphenols production.

Model of the combustion

From the empirical formula of grape pomace ($C_1H_{1.363}N_{0.033}\,O_{0.476}$), which can be determined from its elemental composition (see Table S1) is possible to estimate the mass and energy balances of the combustion process.

The mass balance are carried out from the balance to atoms of carbon (Eq.(S1)), oxygen (Eq.(S2)), hydrogen (Eq.(S3)), and nitrogen (Eq.(S4)). In addition, Eq.(S5) and Eq.(S6) are added to consider that the nitrogen in the air does not react and that a 150% excess of oxygen is considered. This excess air is used to ensure complete oxidation of the compounds used in the reaction and to avoid very high combustor temperatures.

$$z \cdot Nin_{RM} = Nout_{CO_2} \tag{S1}$$

$$\begin{aligned} x \cdot Nin_{RM} + 2 \cdot Nin_{O_2} + Nin_{H_2O} = 2 \cdot Nout_{O_2} + \\ Nout_{H_2O} + 2 \cdot Nout_{CO_2} + 2 \cdot Nout_{NO_2} \end{aligned} \tag{S2}$$

$$y \cdot Nin_{RM} + 2 \cdot Nin_{H_2O} = 2 \cdot Nout_{H_2O} \tag{S3}$$

$$c \cdot Nin_{RM} = Nout_{NO_2} \tag{S4}$$

$$Nin_{N2} = Nout_{N2} \tag{S5}$$

$$Nin_{O2} \cdot 0.4 = NoutO2 \tag{S6}$$

Table S1: Ultimate analysis (wt%) of grape pomace[1].

**Component**	**wt%**
C	47.27
H	5.88
N	1.77
O	45.08

Where z, x, y, c are the coefficients associated with carbon, oxygen, hydrogen and nitrogen respectively. The heat released in the combustion reaction is calculated from the enthalpy of formation (see Table S2) through Eq.(S7). Nin_j and Nout_j are the input and output moles of the combustor of component 'j'.

$$Q_{comb} = \sum_j (AHf_j \cdot Nout_j) - \sum_j (AHf_j \cdot Nin_j) \quad \forall j \in \{j = O_2, H_2O, CO_2 \;\; and \;\; NO_2\} \tag{S7}$$

The combustor is designed so that approximately 40% of the heat is used to produce steam and 60% of the heat is used to heat the flue gas. The final flue gas temperature is calculated by Eqs.(S8)-(S11) while the amount of steam generated is estimated by Eq.(S12).

$$\sum_j (AHf_j \cdot 0.6 \cdot Nout_j + Cpout_j \cdot Nout_j) = \sum_j (AHf_j \cdot 0.6 \cdot Nin_j + Cin_j \cdot Nin_j) \tag{S8}$$

$$Cpout_j = A_j(Tout - Tref) + B_j \cdot \frac{1}{2} \cdot (Tout - Tref)^2 + \\ C_j \cdot \frac{1}{3} \cdot (Tout - Tref)^3 + D_j \cdot \frac{1}{4} \cdot (Tout - Tref)^4 \tag{S9}$$

$$Cpin_j = A_j(Tin - Tref) + B_j \cdot \frac{1}{2} \cdot (Tin - Tref)^2 + \tag{S10}$$

$$C_j \cdot \frac{1}{3} \cdot (Tin - Tref)^3 + D_j \cdot \frac{1}{4} \cdot (Tin - Tref)^4 \tag{S11}$$

$$m_{steam} \cdot \lambda_{H_2O} = \sum_j (AHf_j \cdot 0.4 \cdot Nout_j) - \\ (AHf_j \cdot 0.4 \cdot Nin_j + Cp_j \cdot Nin_j) \tag{S12}$$

Where Tout and Tin are the inlet and outlet temperatures of the combustor, AHf_j is the enthalpy of formation of each compound 'j' and Cp_j is the heat capacity of each compound 'j'. A_j,B_j, C_j, and D_j are empirical coefficient which depend on the chemical properties of each compound[2]. These parameters together with the enthalpy of formation can be found in Table S2.

Table S2: Enthalpy of formation and parameters to calculate the heat capacity of each compound

	AH(kj/mol)	A	B	C	D
CH_4	-74860	19.521	5.21E-02	1.20E-05	-1.13E-08
O_2	0	28.106	-3.68E-06	1.75E-05	-1.07E-08
CO_2	-393770	19.795	7.34E-02	-5.60E-05	1.72E-08
H_2O	-242000	32.243	1.92E-03	1.06E-05	-3.60E-09
N_2	0	31.150	-0.0136	2.68E-05	-1.17E-08
NO_2	33272.8	24.233	48.358E-03	-2.071E-05	29.308E-11

The heat released in combustion is used to feed the hot reservoir of a regenerative Rankine cycle, whose T-S diagram can be seen in Figure S1.

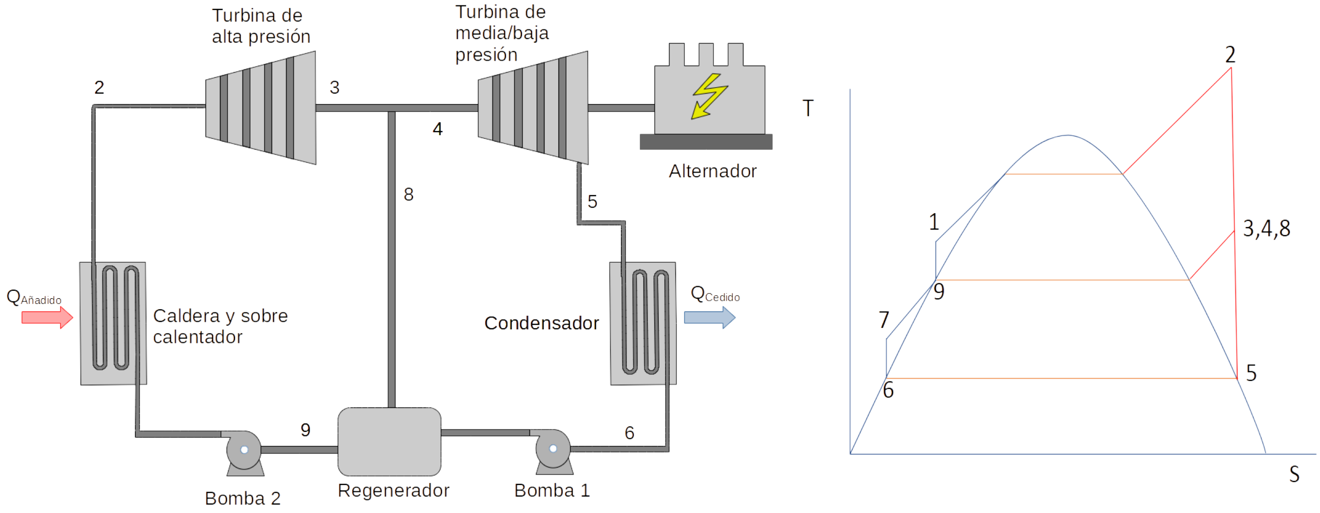

Figure S1: Regenerative rankine cycle

At the hot reservoir of the cycle, a liquid stream (stream 9) is converted into superheated steam (stream 2). Since the temperature and pressure of stream 2 are set at 100 bar and 555K respectively[3], the heat generated in the combustion sets the water mass flow rate of the Ranquine cycle (steam 2).

$$0.4 \cdot Q_{comb} = F2 \cdot (Hwa2 - Hwa1) \tag{S13}$$

The enthalpy of stream 2 can be calculated from the stream pressure and temperature following Eq. (S14)[3].

$$\begin{aligned} Hwa = &(-1.1619E-13 \cdot P^2 - 8.7596E-12 \cdot P- \\ &2.2611E-10)(T)^4 + (4.298E-10 \cdot P^2 + 3.276E-8 \cdot P \\ &+ 7.313E-7) \cdot (T)^3 + (-5.801E-7 \cdot P^2 - 4.6E-5 \cdot P \\ &- 5.009E-4) \cdot (T)^2 + (3.383E-4 \cdot P^2 + 2.947E-2 \cdot P + 2.195) \cdot (T) + \\ &(-7.2042E-2 \cdot P^2 - 7.7877 \cdot P + 2440.8) \end{aligned} \tag{S14}$$

The entropy also depends on these two variables and can be determined from Eq.(S15)[3].

$$
\begin{aligned}
Swa = & (1.5719E-11 \cdot P + 7.4013E-10) \cdot (T)^3 + \\
& (-1.0074E-1 \cdot P^2 - 3.0171E-8 \cdot P - 2.8872E-6) \cdot (T)^2 + \\
& (9.4914E-8 \cdot P^2 + 2.9097E-5 \cdot P + 5.0938E-3) \cdot (T) + \\
& (4.1223E-5 \cdot P^2 - 2.8841E-2 \cdot P + 5.9537)
\end{aligned} \tag{S15}
$$

Since the expansion process in the turbine can be considered isentropic, the temperature in stream 3 can be determined if the pressure of this stream is known. This pressure is fixed at 11 bar[3]. Since the temperature and pressure of stream 3 are known, the enthalpy can be determined following Eq.(S14). With this information, the energy generated in the first turbine can be determined by Eq.(S16).

$$
W_{turbine1} = F_2 \cdot (Hwa_3 - Hwa_2) \tag{S16}
$$

After this first turbine, there is a splitter, and therefore, steams 4 and 8 have the same operating conditions (same entropy and enthalpy). Stream 8 is used to preheat stream 6 before the boiler to increase the Ranquine cycle efficiency, while stream 4 is sent to a second turbine of lower operating pressure. The mass ratio between these two stream is a variable of the mathematical model. To determine the energy produced by the second turbine, a similar procedure is performed as for the first turbine, but with the stream 5 at saturation conditions. The operating pressure is set at 0.08 bar[3].

Between stream 5 and 6 water condensation is produced using cooling water. The mass of cooling water required can be determined from Eq.(S17).

$$
Hwa_6 - Hw_5 = \frac{F_5}{\lambda} \tag{S17}
$$

Model of the gasification process

Following the results of Sanchez and Martin,[4] the indirect gasification, which is composed of a gasifier and a combustor is selected to transform grape pomace into energy. In this type of system, the heat required for gasification is supplied by the combustion of the char formed by the gasification process through a heat transfer media (olivine). The mass ratio between the olivine and the dry biomass is 27. In addition, gasification requires 0.4 kg of steam per kilogram of dry biomass[4]. The operating pressure is set at 1.6 bar[4] while the temperature is a variable of the optimization model. This temperature determines the mole fraction of each component of the syngas (Eqs.(S18)-(S24)).

$$y_{CO,syngas} = 133.46 - 1.029E - 1 \cdot T_{syngas} + 2.8792E - 5 \cdot {T_{syngas}}^2 \tag{S18}$$

$$y_{CO_2,syngas} = -9.5251 + 3.7889E - 2 \cdot T_{syngas} - 1.4927E - 5 \cdot {T_{syngas}}^2 \tag{S19}$$

$$y_{CH_4,syngas} = -13.82 + 4.4179E - 2 \cdot T_{syngas} - 4.6467E - 5 \cdot {T_{syngas}}^2 \tag{S20}$$

$$y_{C_2H_4,syngas} = -38.258 + 5.8435E - 2 \cdot T_{syngas} - 1.9868E - 5 \cdot {T_{syngas}}^2 \tag{S21}$$

$$y_{C_2H_6,syngas} = 11.114 - 1.1667E - 2 \cdot T_{syngas} + 3.064E - 6 \cdot {T_{syngas}}^2 \tag{S22}$$

$$y_{H_2,syngas} = 17.996 - 2.6448E - 2 \cdot T_{syngas} + 1.893E - 5 \cdot {T_{syngas}}^2 \tag{S23}$$

$$y_{C_2H_2,syngas} = -4.3114 + 5.4499E - 3 \cdot T_{syngas} - 1.561E - 6 \cdot {T_{syngas}}^2 \tag{S24}$$

Where $y_{j,syngas}$ is the mole fraction of each component 'j' in the syngas. In addition to the composition of the gas, its amount (mDrySyngas) , as well as the amount of tar (mTar) are also determined from the gasification temperature and equations Eq. (S25) and Eq.(S26).

$$mDrySyngas = 28.993 - 4.3325E - 2 \cdot T_{syngas} - 2.0966E - 5 \cdot {T_{syngas}}^2 \tag{S25}$$

$$mTar = 4.5494E - 2 - 1.9759E - 5 \cdot T_{syngas} \tag{S26}$$

The amount of nitrogen, oxygen and sulfur retained by the char is estimated at 6.6%, 8.3% and 4% of that present in the grape pomace, respectively[4]. The carbon retained by the char is determined from the mass balances of the gasification reactor.

An excess of 150% air, together with char, is fed to the combustor at a temperature of 473k. The heat of combustion of the char is estimated at 25000 kJ/kg[5]. In this case, the composition of the flue gas is determined from the material balances at the combustor, assuming complete oxidation of all reactants. For the syngas purification process, a series configuration of two cyclones and an electrostatic precipitator is used. The efficiency of the cyclones is set at 99% for the carbon, ash and olivine and 99.99%[4] for the electrostatic

precipitator. The olivine lost in the cyclones and in the electrostatic precipitator is replaced.

Once the solids have been removed, the syngas goes through 3 stages to make it suitable to produce power. First, a steam reforming stage is used to transform all the hydrocarbons into H_2 and CO. Subsequently, a FeO bed is used to adsorb the H_2S present in the synthesis gas. Finally, a WGSR allows to adjust the H_2/CO ratio to optimize the combustion process in the Bryton cycle[4].

The reactions considered in the steam reforming process are indicated by Eqs.(S27)-(S29).

$$C_nH_m + nH2O \rightarrow nCO + \left(n + \frac{m}{2}\right) H_2 \tag{S27}$$

$$3H_2 + CO \xleftrightarrow{kp1} H_2O + CH_4 \tag{S28}$$

$$CO + H_2O \xleftrightarrow{kp2} H_2 + CO_2 \tag{S29}$$

All hydrocarbons (C_2H_6,C_2H_4 and C_2H_2), except methane, are completely transformed into CO and H_2. Therefore, both energy and matter balances are modeled from stoichiometric relationships. The conversion of methane is determined from thermodynamic equilibrium, following an empirical relationship (Eq.(S30)-(S31))[4]. These equilibria are controlled by the equilibrium constants kp1 and kp2 which depend on the reaction temperature.

$$kp1 = 10^{\left(-\frac{11650}{T(K)}+13.076\right)} = \frac{P_{CO}\ P_{H_2}^3}{P_{CH_4}P_{H_2O}} \tag{S30}$$

$$kp2 = 10^{\left(-\frac{1910}{T(K)}-1.784\right)} = \frac{P_{CO_2}P_{H_2}}{P_{CO}P_{H_2O}} \tag{S31}$$

The maximum temperature is set at 1600K. To determine the amount of iron needed to remove the SH_2 from the synthesis gas, stoichiometric ratios (Eq.(S32)) and 100% conversion are used[4].

$$ZnO + H_2S \rightarrow H_2O + ZnS \tag{S32}$$

A WGSR is used to adjust the H2/CO ratio to an optimal value to maximize energy production. Eq.(S30)-(S31) are used for the composition of the flue gas. The gas is compressed to 4.5 bar, which causes an increase in gas temperature, according to the Eq.(S33)

$$T_{out} = T_{in} + T_{in} \cdot (P_{out}/P_{in})^{((z-1)/z)-1} \cdot \frac{1}{nc} \tag{S33}$$

Therefore, it is necessary to cool it down to 25ºC to feed the PSA tower. This causes the condensation of most of the water contained in this stream. The amount condensed is determined from Rault, Dalton and Antony's law. A PSA system is used to retain the remaining moisture, CO and up to 95% of the CO_2[3]. Once the synthesis gas is purified, it is used to produce electrical energy from a Brayton cycle, whose modeling is shown in 'Model of the anaerobic digestion process'.

Model of the anaerobic digestion process

The feed stream is mixed with a water stream until a solids concentration of 10% is reached. Most of the water used in this stage is recovered at the end of the process, in the dehydration

of the digestate. This not only saves water, but it also helps the anaerobic digestion process by acting as an inoculum for the biological reaction. The output stream is heated to 37ºC ($T_{digestion}$) to achieve the optimal digestion conditions[6]. The mass and energy balances are shown by Eqs.(S34)-(S36).

$$f_{H_2O,feed} = (f_{Lipids,feed} + f_{Ch,feed} + f_{Prot,feed} + f_{Ash,feed} + f_{NH_3,feed} + f_{Rest,feed}) \cdot 0.10 \tag{S34}$$

$$Q_{preheating} = F_{steam1} \cdot \lambda_{steam} \tag{S35}$$

$$Q_{preheating} = F_{feed} \cdot Cp_{H2O} \cdot (T_{digestion} - Tref) \tag{S36}$$

Where $f_{lipids,feed}$,$f_{ch,feed}$,$f_{Prot,feed}$,$f_{Ash,feed}$, and $f_{NH_3,feed}$ are the mass flows of lipids, carbohydrates, protein, ash and inorganic nitrogen in the feed, while $f_{rest,feed}$ is the non-reaction biomass of the waste, λ_{steam} is the heat of vaporization of the steam and Cp_{H2_2O} is the heat capacity of the water. It is considered that the heat capacity of the mixer output stream will be similar to the heat capacity of the water since 90% of this stream is water. Both variables depend on the temperature, as can be seen in the literature[2]. T_{ref} is the reference temperature (25ºC) and F_{feed} is the total feed mass.

For the modeling of anaerobic digestion process of the grape pomace, the model present in the work of Taifouris and Martin[7] is used. For this purpose, the stoichiometric ratios of fats, proteins and carbohydrates are used, following Eqs.(S37)-(S40) and Eqs.(S44-S46).

$$f_{CH_4,digestor} = 0.6588 \cdot fLir + 0.2433 \cdot fCHr + 0.2383 \cdot fPrr \tag{S37}$$

$$f_{CO_2,digestor} = 0.6644 \cdot fLir + 0.6675 \cdot fCHr + 0.6822 \cdot fPrr \tag{S38}$$

$$f_{NH_3,digestor} = 0.0280 \cdot fLir + 0.0227 \cdot fCHr + 0.1647 \cdot fPrr \tag{S39}$$

$$f_{H_2O,digestor} = -0.4810 \cdot fLir - 0.039 \cdot fCHr - 0.2047 \cdot f\Pr r \tag{S40}$$

Eq.(S41) determines the amount of cell mass that is generated in the process.

$$f_{bat,digestor} = 0.1857 \cdot fLir + 0.1508 \cdot fCHr + 0.1241 \cdot f\Pr r \tag{S41}$$

Therefore, the mass of the biogas and the digestate is calculated by Eqs.(S42)-(S43)

$$F_{biogas} = f_{CH4,digestor} + f_{CO2,digestor} + f_{NH3,biogas} + f_{H2O,biogas} \tag{S42}$$

$$F_{digestate} = (f_{lipids,feed} + f_{ch,feed} + f_{prot,feed}) \cdot 0.5 + f_{rest,feed} + f_{NH3,digestate} + f_{H2O,digestate} \quad \text{(S43)}$$

From experimental data obtained from grape pomace biodegradability, an average biodegradability of 0.5[8–13] is considered for all compounds (Eqs.(S44)-(S46)).

$$fCHr = f_{ch,feed} \cdot 0.5 \quad \text{(S44)}$$

$$fLir = f_{Li,feed} \cdot 0.5 \quad \text{(S45)}$$

$$f\,\mathrm{Pr}\,r = f_{Prot,feed} \cdot 0.5 \quad \text{(S46)}$$

The thermodynamic equilibrium is used to estimate the distribution of H_2O and NH_3, between the gas and liquid phases (the rest of the gases can be considerded insoluble according to Henry's constants[2]), by using the laws of Dalton, Raoult and Antoine (Eqs.(S47)-(S51)).

$$y_{NH_3,biogas} = Pv_{NH_3} \cdot x_{NH_3,digestate} \quad \text{(S47)}$$

$$y_{H_2O,biogas} = \frac{MW_{H_2O}}{MW_{biogas-dry}} \cdot \frac{Pv_{H_2O}}{Pt_{Digestion} - Pv_{H_2O}} \quad \text{(S48)}$$

$$MW_{biogas-dry} = \sum_i y_{i,biogas} \cdot MW_i \quad \forall i \in \{i = CH_4, CO_2, NH_3\} \tag{S49}$$

$$\sum_i x_{i,digestate} = 1 \quad \forall i \in \{i = bat, lip, ch, prot, lig, ash, NH_3, H_2O, rest\} \tag{S50}$$

$$\sum_i y_{i,biogas} = 1 \quad \forall i \in \{i = CH_4, CO_2, NH_3, H_2O\} \tag{S51}$$

Where $y_{j,biogas}$ is the mole fraction of component 'j' in the biogas (gas phase) and $x_{j,biogas}$ is the mole fraction of component 'j' in the digestate (liquid phase). MW_i is the molecular mass of component 'i' (CH_4,CO_2,NH_3,Biogas or Water). Pv_{H_2O} is the vapor pressure of the water and Pt is the operating pressure of the digestion, which is , one bar. Once the mole fractions of water and ammonia in the liquid and gas phase are calculated, and the total mass of each of the phases is known, the amount of each of these chemical compounds in both phases can be determined (Eqs.(S52)-(S57)).

$$f_{NH3,biogas} = y_{NH_3,biogas} \cdot N_{biogas} \cdot MW_{NH_3} \tag{S52}$$

$$f_{NH3,digestate} = x_{NH_3,digestated} \cdot N_{digestate} \cdot MW_{NH_3} \tag{S53}$$

$$f_{H_2O,biogas} = y_{H_2O,biogas} \cdot N_{biogas} \cdot MW_{H_2O} \tag{S54}$$

$$f_{H_2O,digestate} = x_{H_2O,digestate} \cdot N_{digestate} \cdot MW_{H_2O} \tag{S55}$$

$$N_{biogas} = \sum_i \frac{f_{i,biogas}}{MW_i} \quad \forall i \in \{i = CH_4, CO_2, NH_3, H_2O\} \tag{S56}$$

$$N_{digestate} = \sum_i \frac{f_{i,digestate}}{MW_i} \quad \forall i \in \{i = bat, lip, ch, prot, lig, ash, NH3, H2O, rest\} \tag{S57}$$

Where N$_{biogas}$ is the total moles of the gas stream (biogas), and N$_{digestate}$ are the total moles of the liquid stream (digestate). The volume of biogas (V$_{biogas}$), assuming ideal gas, is estimated by Eq.(S58)

$$V_{biogas} = \frac{0.082\frac{atm \cdot l}{mol \cdot k} \cdot 310K \cdot (\sum_i \frac{m_{i,biogas}}{MW_i})}{Pt_{Digestion}} \forall i \in \{i = CH_4, CO_2, NH_3, H_2O\} \tag{S58}$$

The composition of the digestated is estimated through a mass balance between the compounds that enter the digester with the raw material (see Table S3) and those that leave this equipment with the biogas (Eqs(S59)-(S65)).

Table S3: Composition of the Grape pomace (RM: raw material)

	g/KgRM	References
**TS**	434	14
**VS**	394	
**Total Nitrogen**	7	
**Phosphorous**	2	
**Water**	566	
**Lipids**	62	15
**Carbohydrates**	220	
**Protein**	107	
**N-NH4+**	0.4	16
**Potasium**	13	17
**Polyphenols**	32	18
**Tannis**	96	19

$$f_{C,digestate} = f_{C,feed} - f_{CH_4,biogas} \cdot \frac{MW_C}{MW_{CH_4}} - f_{CO_2,biogas} \cdot \frac{MW_C}{MW_{CO_2}} \tag{S59}$$

$$f_{N_{org},digestate} = f_{Norg,feed} \tag{S60}$$

$$f_{N_{am},digestate} = f_{Nam,feed} - f_{NH3,biogas} \cdot \frac{MW_N}{MW_{NH3}} \tag{S61}$$

$$f_{P,digestate} = f_{P,feed} \tag{S62}$$

$$f_{K,digestate} = f_{K,feed} \tag{S63}$$

$$f_{REST,digestate} = f_{REST,feed} \tag{S64}$$

$$f_{H2O,digestate} = f_{H2O,feed} - F_{biogas} \cdot y_{H_2O,biogas} \tag{S65}$$

The heat required to maintain the anaerobic digestion reaction and keep the reactants in mesophilic conditions is determined from an empirical factor[20] which relates the heat required to the volatiles solids reacting (3.6 kJ per gram of volatile solid degraded).

The biogas must be upgraded before being fed to the Brayton cycle. First, the H_2S must be removed. For this, an iron oxide bed is used, which reacts with H_2S producing produces iron sulfide and water, assuming 100% conversion[4]. A PSA system with a 5A zeolite packed bed is used to completely remove CO, NH_3, and H_2O. 95% of the CO_2 is also retained[3]. This process is carried out at a pressure of 4.5 bar and 25ºC. The digestate is dehydrated by using a filter, dried up to 10% moisture and stored[6].

A Brayton cycle is proposed to produce energy from the combustion of the biomethane produced. The use of a combined cycle is discarded since the combustion gases are to be used to supply the energy needed for the digestion process. The combustion process is modeled from stoichiometric ratios based on methane combustion, considering a 100% conversion. A 150% excess of air with respect to the stoichiometric ratio is used (Eq.(S66)). The atomic balances, explained at the beginning of 'Model of the combustion', can be used to estimate the balance of matter in this combustion process.

$$CH_4 + 2O_2 \rightarrow CO_2 + 2H_2\ O \tag{S66}$$

Since this is a combustion process, the energy balances shown in 'Model of the combustion' can be used to estimate the flue gas outlet temperature, but taking into account that in this case methane is combusted.

On the one hand, the electrical energy needed to compress the biogas before the combustion process and that produced in the turbine, due to the expansion of the combustion

gases, is determined from Eq.(S67).

$$W = F + \frac{R \cdot z \cdot T_{in}}{MW \cdot (k-1)} \cdot \left(\frac{P_{out}}{P_{in}}\right)^{\frac{(k-1)}{k}-1} \cdot \frac{1}{nc} \tag{S67}$$

Where k is the polytropic coefficient (1.4), nc is the compressor or turbine efficiency (0.85). T_{in} is the inlet temperature, P_{out} is the inlet pressure, and P_{in} is the inlet pressure.

On the other hand, the temperature of the gases at the outlet of the compressors and the turbines is calculated from Eq.(S68)

$$T_{out} = T_{in} + T_{in} \cdot (P_{out}/P_{in})^{((z-1)/z)-1} \cdot \frac{1}{nc} \tag{S68}$$

The turbine exhaust gases are used to supply heat to the bioreactor.

Model of the pyrolisis

Due to the large number of chemical and physical reactions that occur in the pyrolysis process of a waste, we proceeded to model them using empirical yields from the literature. According to the work of Ateş et al[1] for the pyrolysis of 1 kg of dried grape pomace, 0.31 kg of biochar, 0.31 kg of bio-oil and 0.38 kg of gas are generated. The composition of each of the phases is also estimated from empirical data[1].

To perform the pyrolysis process it is necessary to heat the raw material to the optimum reaction conditions, that is, 500ºC and to supply the heat necessary to carry out the chemical and physical reactions and maintain the isothermal system, since the process is endothermic. For this purpose, the flue gases from the bio-oil combustion process and the gas produced in the pyrolysis are used. In this way, the process is auto-thermal. The energy balance that determines the flue gas outlet temperature is shown by Eq. (S69)

$$\sum_j (CpFG_j \cdot NFG_j) = \sum_j (CpRM_j \cdot NRM_j) + Qpyro \tag{S69}$$

Where $CpFG_j$ the integration of the specific heat variations between the inlet and outlet temperatures of the flue gas (Eq. (S69)), $CpRM_j$ is the integration of the specific heat variations between the inlet and outlet temperatures of the raw material (Eq. (S69)), while NFG_j and NRM_j are the moles of the flue gas and raw material, respectively. The heat required to keep the reaction isothermal (Qpyro) is estimated from the work of Xu et al[21] and its value is 1930 J/g biomass feed.

$$\begin{aligned} &CpFG_j = A_j(TFGout - TFGin) + B_j \cdot \frac{1}{2} \cdot (TFGout - TFGin)^2 + \\ &C_j \cdot \frac{1}{3} \cdot (TFGoutTFGin)^3 + D_j \cdot \frac{1}{4} \cdot (TFGout - TFGin)^4 \end{aligned} \tag{S70}$$

$$\begin{aligned} &CpRM_j = A_j(TRMout - TRMin) + B_j \cdot \frac{1}{2} \cdot (TRMout - TRMin)^2 + \\ &C_j \cdot \frac{1}{3} \cdot (TRMout - TRMin)^3 + D_j \cdot \frac{1}{4} \cdot (TRMout - TRMin)^4 \end{aligned} \tag{S71}$$

Where A_j,B_j, C_j and D_j are empirical factors to estimate the specific heat of the raw material and depend on the compound 'j' of the grape pomace.

The gas and bio-oil are used to produce energy to dry the raw material entering the process. For this, it is necessary to determine the amount of energy that can be obtained from these products, via combustion. The composition of the gas as well as the elemental composition of the bio-oil are shown in the following tables (S4) y (S5) respectively.

Table S4: Composition of the gas generated by the pyrolisis of the grape pomace[1]

**Component**	**wt%**
H2	8.00
CH4	8.00
CO	28.00
CO2	56.00

Table S5: Ultimate analysis of bio-oils generated by the pyrolisis of the grape pomace[1].

**Component**	**wt%**
C	69.28
H	7.82
N	2.33
O	20.57

On the one hand, from the elemental composition, it is possible to obtain the empirical formula of bio-oil, which is as follows $CH_{1.33}O_{0.179}N_{0.032}$. Using the procedure described in 'Model of the combustion', it is possible to determine the amount of energy that can be released in the bio-oil combustion process (in this case all the heat goes to heat the flue gas). On the other hand, from the composition of the gas, the energy released in the combustion process can be determined, following the procedure described in 'Model of the anaerobic digestion process' for the synthesis gas, but taking into account that this gas, in addition to methane, also contains H_2 and CO. Therefore, two more oxidation reactions should be added. (Eqs.(S72)-(S73)).

$$H_2 + 1/2O_2 \rightarrow 2H_2\,O \tag{S72}$$

$$CO + 1/2O_2 \rightarrow CO_2 \tag{S73}$$

These gases are combined and the equilibrium temperature is determined from energy balances (Eq.S74).

$$\sum_j (Cpout_j \cdot Nout_j) = \sum_j (Cpin1_j \cdot Nin1_j + Cpin1_j \cdot Nin2_j) \quad \text{(S74)}$$

Where Nin1_j and Nin2_j are the moles of each compound 'j' of each input stream (gases from the combustion of bio-oil and gases from the combustion of the gas generated in pyrolysis), while that Nout_j are the moles of each compund 'j' of of the mixer outlet gas. Cpin1_j, Cpin2_j and Cpout_j are the specific heats of the input and output streams and are calculated from Eqs.(S75-S77)

$$\begin{aligned} &Cpout_j = A_j(Tout - Tref) + B_j \cdot \frac{1}{2} \cdot (Tout - Tref)^2 + \\ &C_j \cdot \frac{1}{3} \cdot (Tout - Tref)^3 + D_j \cdot \frac{1}{4} \cdot (Tout - Tref)^4 \end{aligned} \quad \text{(S75)}$$

$$\begin{aligned} &Cpin1_j = A_j(Tin1 - Tref) + B_j \cdot \frac{1}{2} \cdot (Tin1 - Tref)^2 \\ &+ C_j \cdot \frac{1}{3} \cdot (Tin1 - Tref)^3 + D_j \cdot \frac{1}{4} \cdot (Tin1 - Tref)^4 \end{aligned} \quad \text{(S76)}$$

$$\begin{aligned} &Cpin2_j = A_j(Tin2 - Tref) + B_j \cdot \frac{1}{2} \cdot (Tin2 - Tref)^2 + C_j \cdot \\ &\frac{1}{3} \cdot (Tin2 - Tref)^3 + D_j \cdot \frac{1}{4} \cdot (Tin2 - Tref)^4 \end{aligned} \quad \text{(S77)}$$

The resulting gas is used to dry the feedstock since pyrosilis requires the feedstock to be dried (10% moisture) and grape pomace contains slightly more than 50% moisture[22].

Integrated multyproduct system

The integrated multi-product system is composed of 3 different process lines, the production of oil, polyphenols and biochar. It is an integrated system in which the residues from one

process are used to produce a new product in the next line. Thus, polyphenols are produced from the residues of the oil production line, while biochar is produced from the residues of the polyphenol production process. The process is modeled by means of material and energy balances that can be derived from the work of Jin et al.[18]

Production of oil

First, the feedstock is dried using flue gas generated from the combustion of part of the grape pomace. This stage is modeled following the procedure described in 'Drying of the raw material'. Subsequently, the seeds are separated from the skins. The separation ratio between seeds and skins is 16:9. The seeds are sent to the extration process to obtain their oil. The first step is to heat the inlet stream up to the extraction temperature, that is 60 °C. For this purpose, a steam, which is generated together with the flue gas in the combustion of the grape pomace, is used.

The energy balance is indicated by Eq.(S78).

$$\begin{aligned} & m_{steam} \cdot \lambda_{H_2O} = Fseed \cdot x_{seed} \cdot (A_j \cdot (Tseedout - Tseedin) \\ & + 1/2 \cdot B_j \cdot (Tseedout^2 - Tseedin^2) + \\ & 1/3 \cdot C_j \cdot (Tseedout^3 - Tseedin^3) + 1/4 \cdot D_j \cdot (TFout^4 - Tseedin^4)) \end{aligned} \tag{S78}$$

Where Fseed is the flow rate of the stream containing the seeds, while x_{seed} is the composition of the seeds in terms of lipids, carbohydrates, protein, fiber and ash. The terms A_j,B_j,C_j and D_j are the same as those used for drying grape pomace in 'Drying of the raw material'. Tseedout is 60°C while $T_{seeedin}$ is 25°C.m_{steam} is the mass of vapor and λ_{H2O} is the latent heat of water.

The oil extraction process is carried out by hexane, at a ratio of 3:1 with respect to the stream containing the seed. From the equipment design data presented in the supplementary material to the work of Jin et al,[18] the ratio of the input to output streams can be deduced.

By analyzing the total concentration of oil in the seed, the stream ratio and the extraction yield (0.987), it is determined that up to 0.146 kg of oil can be obtained per kg fed to the extractor. From the annual hexane consumption presented in the results of the Jin et al[18] work, it can be determined that the amount of hexane to be introduced into the process is 0.0057kg per kg of raw material. Most of the hexane is recovered in the solvent recovery process. 0.6714 kg of water is lost per kg of hexane. From the oil extracted and the amount of hexane lost, the amount of residue generated at this stage is computed using a global mass balance in the extractor. This residue is mixed with the skins and sent to the polyphenol production line.

The oil stream is heated up to 80ºC following a procedure similar to the one indicated in Eq.(S78). But in this case, only parameters A,B,C,D corresponding to lipids are considered. Subsequently, the oil is washed with water (0.3kg of H_2O per kg of oil) and NaOH (0.2kg per kg of oil). 16.83% of the scrubber output stream is separated as solid residue. The remaining residue is separated after the second wash. This second wash is performed with water (0.1 kg of H_2O per kg of oil) at 60ºC. The stream containing the oil is dried following the procedure described in 'Drying of the raw material' and fed to the bleaching tank, where 0.03 kg of clay is fed per kg of oil. A filter is used to remove the spent clay (1.036 kg of spend clay is removed per kg of clay added to the system). Finally, the oil is subjected to a deodorization process by increasing its temperature up to 230ºC. This process is carried out by a fire heater fed with part of the raw material. To determine the amount of grape pomace to be combusted, Eq. (S79) is used.

$$\begin{aligned} f_{GP} \cdot Qcomb = F_{oil} \cdot (A_{oil} \cdot (230 - 115) + 1/2 \cdot B_{oil} \cdot (230^2 - 115^2) \\ +1/3 \cdot C_{oil} \cdot (230^3 - 115^3) + 1/4 \cdot D_{oil} \cdot (230^4 - 115^4)) \end{aligned} \tag{S79}$$

Where Qcomb is the heat of combustion which can be determined using the procedure described in 'Model of the combustion' while f_{GP} is the mass of grape pomace used as fuel.

Production of polyphenols

The residues proceeding from the oil extraction line are heated up to the optimum extraction conditions, that is, up to 70ºC. The energy balances are similar to those shown by Eq.(S78). In this case, the extraction is carried out through a 40% ethanol solution. The ethanol must also be heated to 70ºC. The ethanol feed ratio is 5:1 with respect to the waste feed. Through the equipment design of the supplementary material of the work of Jin et al[18] , it is possible to determine the amount of material that goes to the evaporator and the amount that goes to the mixer. The 63.67% of the extractor output stream continues the polyphenol production line, as it is the polyphenol-rich stream, while the residual stream is sent to a mixer, where it will join the residue from the next extraction.

The polyphenol-rich stream is sent to an evaporator to recover part of the ethanol used. After the evaporation process, 94.1% of the total stream is evaporated (this stream is considered to contain only ethanol and water). It is also known that the non-evaporated part has been enriched by 65% in solid components, so that the remaining 35% is water.

The global balance at the evaporator, which is necessary to determine the mass of steam needed for the vaporization process, is shown by Eq.(S80).

$$\begin{aligned}&(Qoutgas + Qoutliq + Qpoly) - (Qinliq) = \\ &m_{steameva1} \cdot 525.7kcal/kg \cdot 4.18kj/kcal\end{aligned} \tag{S80}$$

Where Qoutgas, Qoutliq and Qinliq are the enthalpy of the outlet gaseous stream, outlet liquid stream and inlet liquid stream, respectively. The enthalpy calculation of each of these streams is shown by Eq.(S81)-(S83). The evaporator outlet temperature (97ºC) can be estimated from the composition of the gaseous and liquid streams, assuming an ethanol-water system reaching thermodynamic vapor-liquid equilibrium.

$$Qoutgas = \sum_j Foutgas_j \cdot (A_j \cdot (Tout - Tref) + 1/2 \cdot B_j \cdot (Tout^2 - Tref^2) + \\ 1/3 \cdot C_j \cdot (Tout^3 - Tref^3) + 1/4 \cdot D_j \cdot (Tout^4 - Tref^4)) \tag{S81}$$

$$Qoutliq = \sum_j Foutliq_j \cdot (A'_j \cdot (Tout - Tref) + 1/2 \cdot B'_j \cdot (TEout^2 - Tref^2) + \\ 1/3 \cdot C'_j \cdot (TEout^3 - Tref^3) + 1/4 \cdot D'_j \cdot (TFout^4 - Tref^4)) \tag{S82}$$

$$Qinliq = \sum_j Fin_j \cdot (A'_j \cdot (Tin - Tref) \cdot +1/2 \cdot B'_j \cdot (Tin^2 - Tref^2) + \\ 1/3 \cdot C'_j \cdot (Tin^3 - Tref^3) + 1/4 \cdot D'_j \cdot (Tin^4 - Tref^4)) \tag{S83}$$

Parameters A,B,C,D are the empirical parameters related to the specific heat of gases while A',B',C', and D' are the parameters related to the specific heat of liquids. These empirical parameters can be found in the literature[2]. Qpoly is the heat absorbed by the polyphenols, which is determined by the correlation shown by Eq.(S84)[23].

$$Qpoly = (0.1243 \cdot Tout + 303.69) \cdot Finpoly/138.12 \cdot (Tout - Tin) \tag{S84}$$

In this second extraction step, a 95% ethanol stream is used to purify the polyphenol stream. In this case, 0.5 kg of ethanol is added per kg of feed. From the extractor output, 56.8% continues with the polyphenol production line while the rest is considered waste and sent to a mixer, where it is mixed with the residual output of the first extractor. This purification process allows 65% of the impurities to be precipitated[18]. As the total amount of polyphenols is known (0.0319 kg per kg of dried grape pomace) and also its extraction yield (82.8%), the amount of impurities in the stream can be determined. Since this piece

of equipment is a splitter, water and ethanol are considered to be distributed in the same proportion in the solid phase as in the liquid phase, in order to reduce the degrees of freedom in determining the composition of the streams.

The polyphenol-rich stream is fed to an evaporator to recover the ethanol used. The 58.59% of the fed stream is sent to a dryer (polyphenol-rich stream) and the rest (ethanol and water) are mixed with the gaseous output of the previous evaporator. Mass balances to this evaporator are modeled like the previous evaporator. The polyphenols must be dried to 7% moisture. The drying process is modeled as indicated in 'Drying of the raw material'. The polyphenol-rich dryer output stream constitutes 73.72% of the dryer feed stream, while the remainder is the matter removed by the flue gas.

The residues from the extractions are sent to a desolventizer to recover ethanol. The yield of the desolventizing process is 80%. After this step, 34% of the input stream is evaporated, while the rest, composed of solid residue, is sent to a drying process. All gaseous streams of water and ethanol are blended and sent to the ethanol recovery line. These streams are cooled using cooling water and then distilled to obtain a 95% ethanol stream. A portion of this stream is used for the purification stage of the polyphenol extraction process, while the remainder is diluted with water to 40% to extract the polyphenols.

Production of biochar

After the desolventizing process, the residues are cooled from 89°C to 60°C, dried to a humidity of 2% and fed to the pyrolysis reactor. The pyrolysis process is modeled as indicated in 'Model of the pyrolisis'.

Drying of the raw material

For the combustion, gasification, pyrolysis and IMPS processes it is necessary to dry the feedstock.

For this, either the products of the process are used to generate heat (auto-thermal

process) or part of the grape pomace is combusted to utilize the heat generated. A priori it is not known exactly which processes are autothermal and which are not, and therefore, in all drying processes the possibility of using a certain amount of raw material as fuel is considered.

The raw material outlet conditions are set at 50ºC and 10% humidity for all processes. Therefore, the temperature of the flue gas can not be lower than 60ºC and its maximum relative humidity cannot exceed 90%.

The mass balances are shown by the Eq.(S85)-(S87).

$$F_{FG_{in}} = F_{FG_{out}} \quad j \neq H_2O \tag{S85}$$

$$F_{RMin} = F_{RMout} \quad j \neq H_2O \tag{S86}$$

$$F_{FG_{out}} = F_{FGin_j} + (F_{RMout_j} - F_{RMin_j}) \;\; j = H_2O \tag{S87}$$

The general energy balance in the drying process is shown by Eq.(S88).

$$QinFG + QinRM = QoutRM + QoutFG \tag{S88}$$

The raw material comes into contact with the flue gas, increasing its temperature up to a maximum of 50ºC. It is considered that only water is removed from the grape pomace. The energy balances at the inlet and outlet of the dryer applied to the grape pomace (QinFG and QoutFG, respectively) are shown by Eqs.(S89)-(S90).

$$
\begin{aligned}
&QinRM = xin_j \cdot FRMin \cdot (A_j \cdot (TRMin - TRMref) + \\
&B_j \cdot 1/2 \cdot ((TRMin - 273)^2 - (TRMref - 273)^2) - \\
&C_j \cdot 1/3 \cdot ((TRMin - 273)^3 - (Tref - 273)^3)) \;\; j = H2O
\end{aligned} \tag{S89}
$$

$$
\begin{aligned}
&QoutRM = xout_j \cdot FRMout \cdot (A_j \cdot (TRMout - Tref) \\
&+ B_j \cdot 1/2 \cdot ((TRMout - 273)^2 - (Tref - 273)^2) - \\
&C_j \cdot 1/3 \cdot ((TRMout - 273)^3 - (Tref - 273)^3)) \;\; j = H2O
\end{aligned} \tag{S90}
$$

The parameters A_j,B_j,and C_j are empirical parameters that can be consulted in the literature[24] and depend on the type of grape pomace component (protein, carbohydrate, lipids, etc). TRMin and TRMout are the inlet and outlet temperature of the grape pomace, respectively. FRMin and FRMout are the grape pomace inlet and outlet mass flow rates. Finally, xin_j and $xout_j$ are the composition of grape pomace (lipids, carbohydrates, protein, etc.) at the inlet and outlet.

The mass balances of the flue gas, inlet and outlet of the dryer are shown by the Eq.(S91)-(S92).

$$
\begin{aligned}
&QFGin = A'_j \cdot (TFGin - Tref)) + ((1/2) \cdot B'_j \cdot (TFGin^2 - Tref^2)) + \\
&((1/3) \cdot C'_j \cdot (TFGin^3 - Tref^3)) + ((1/4) \cdot D'_j \cdot (TFGin^4 - Tref^4)) \cdot NFGinj
\end{aligned} \tag{S91}
$$

$$
\begin{aligned}
&QFGout = A'_j \cdot (TFGout - Tref) + ((1/2) \cdot B'_j \cdot (TFGout^2 - Tref^2)) \\
&+ ((1/3) \cdot C'_j \cdot (TFGout^3 - Tref^3)) + ((1/4) \cdot D'_j \cdot (TFGout^4 - Tref^4)))) \\
&\cdot (xout_{H2O} \cdot FRMout - xin_{H2O} \cdot FRMin) \cdot \lambda_{H_2O}
\end{aligned} \tag{S92}
$$

The parameters A'_j,B'_j,and C'_j, D'_j are empirical parameters associated with each com-

ponent of the flue gas (CO_2,N_2,O_2, or H_2O) and can be found in the literature[2]. TFG_{in} and TFG_{Out} are the flue gas inlet and outlet temperature, respectively. $NFGin_j$ and $NFGout_j$ are the moles of each flue gas component at the inlet and outlet of the dryer.

For the gas used to dry the grape pomace, two requirements must be met, its temperature must not fall below 60°C and its relative humidity (ϕ) must not exceed 90%. To determine the relative humidity, the Eqs.(S93)-(S95) are used. This is obtained by combining Raoult's, Antoine's and Dalton's law, as well as the ideal gas equation.

$$Pa = \frac{MWgas}{18 \cdot \sum_j F_{FG_j} - F_{FG_{H_2O}}} \cdot (Pt - Pa) \cdot (F_{FG_{H_2O}}) \tag{S93}$$

$$Ps = 10^{A - \frac{B}{T(C)+C}} \tag{S94}$$

$$\phi = \frac{Pa}{Ps} \tag{S95}$$

Those processes that do not meet these two requirements must use another heat source to dry the raw material. To consider these cases, the Eq.(S88) is modified to Eq.(S96).

$$QinFG + QinRM = QoutRM + QoutFG + Qextra \tag{S96}$$

Where Qextra is the heat required to achieve the drying conditions. This heat is minimized by adjusting the flue gas exit conditions to the more restrictive of the two conditions presented above.

$$\begin{aligned} Qextra = \; & Fextra \cdot (A'j \cdot (TEout - TEin) + 1/2 \cdot B' \cdot (TEout^2 - TEin^2) + \\ & 1/3 \cdot C' \cdot (TEout^3 - TFGin^3) + 1/4 \cdot D' \cdot (TFout^4 - TFGin^4)) \end{aligned} \tag{S97}$$

Fextra is the amount of flue gas required to complete the drying process, which is generated with a part of the feedstock, from the combustion process described in 'Model of the combustion'. TEout and TEin are the outlet and inlet temperatures of this flue gas.

Economic, environmental and social impact estimation of each process

OPEX estimation of the processes considered

The prices of the products obtained through the processes considered, as well as the cost of raw materials, energy and utilities can be seen in the Table S6.

Table S6: Prices of the product, raw material and utilities used.

**Product**	**Value**	**Reference**
Power($/kwh)	0.10	[25]
Tannins (US-$/kg)	1.50	[26]
Fertilizer (US-$/kg)	2.47	[27]
Biochar (US-$/kg)	2.47	
Oil (US-$/kg)	4.00	
Polyphenols (US-$/kg)	20.00	
**Raw Material**	**Value**	**Reference**
Grape Pomace (US-$/metric ton)	32.00	[27]
NaOH (US-$/kg)	0.41	
Etanol (US-$/kg)	0.78	
Hexane (US-$/kg)	0.9	
Water (US-$/metric ton)	0.35	
Clay (US-$/kg)	0.35	
Na_2SO_3		[28]
**Utility**	**Value**	**Reference**
cooling water (US-$/metric ton)	0.05	[27]
steam (US-$/metric ton)	17.00	

Environmental impact estimation of the processes considered

To estimate the environmental impact, the carbon footprint generated in each process is evaluated by analyzing gas and solid emissions. For this purpose, the CO_2 equivalent is used. The equivalences can be seen in Table S7.

CAPEX estimation of the processes considered

It it necessary to calculate the CAPEX of the factory to estimate the fixed operating cost[30]. CAPEX is estimated following different procedures depending on each process:

Table S7: CO_2 equivalences[29]

Chemical (kg)	CO_2eq (kg)
CO_2	1
NH_3	2.11
Ethanol	1.24
Solid Waste	1.47E-02
Steam	0.61
Soap	1.75
Water	3.00E-05

- Combustion and tannin production process: The cost of each piece of equipment is estimated using the empirical correlations shown in the work of Couper et al.[31]

- Gasification: The reactors for the water gas shift reaction, ZnO absorption, steam reforming and gasification process are estimated following the methodology shown in the work of Sanchez and Martin.[32] The cost estimation of filters, cyclones and electric precipitator is developed following the work of Almena and Martin.[33] The rest of the equipment, such as heat exchangers, compressor or furnaces, are estimated following the work of Couper et al.[31]

- Anaerobic Digestion: The cost of the digester is calculated following the cost estimation described in the work of Taifouris and Martin.[34] All other equipment is estimated following the work of Couper et al.[31]

- Pyrolisis: The cost is estimated from the plant capacity and the empirical correlation (Eq.(S98)) from the work of Ramos and Ferreira.[35]

$$CAPEX_{pyrolisis}(€) = 1973.25 \cdot F\left(\frac{drykg}{h}\right) \cdot \left(\frac{CEPCI_{2023}}{CEPCI_{2022}}\right) \tag{S98}$$

- IMPS: Both for the single oil production and for the process that also integrates the production of polyphenols and biochar, the procedure used is described in the work

of Jin et al[36] and consists of a capacity ratio (Eq.(S99)) for the oil production and (Eq.(S100)) for the polyphenols production .

$$CAPEX_{oil}(MMe) = 14.6 \cdot \left(\frac{F(t/year)}{32659}\right)^{0.6} \cdot \left(\frac{CEPCI_{2023}}{CEPCI_{2021}}\right) \tag{S99}$$

$$CAPEX_{polyphenols}(MMe) = 37.7 \cdot \left(\frac{F(t/year)}{32659}\right)^{0.6} \cdot \left(\frac{CEPCI_{2023}}{CEPCI_{2021}}\right) \tag{S100}$$

Result

The results of the material balances, as well as the investment cost (CAPEX) and the operational costs (OPEX) of each process can be found in the Tables S8-S9 of the supplementary material.

Table S8: Production and consumption of added-value products, raw materials, services, wastes, and energy

		**Combustion**	**Gasification**	**Anaerobic Digestion**	**Pyrolisis**	**Tannins E**	**Oil E.**	**IMPS**
		**Raw Material**						
GP burnet	kg/kgGDP	0.000	0.000	0.000	0.426	0.000	0.138	0.400
Water		0.000	0.474	6.172	0.000	33.814	0.020	0.231
Na_2SO_3		0.000	0.000	0.000	0.000	0.025	0.000	0.000
Olivine		0.000	0.006	0.000	0.000	0.000	0.000	0.000
NaOH		0.000	0.000	0.000	0.000	0.025	1.01E-04	0.000
Ethanol		0.000	0.000	0.000	0.000	0.000	0.000	0.022
Hexano		0.000	0.000	0.000	0.000	0.000	0.002	0.002
Clay		0.000	0.000	0.000	0.000	0.000	0.002	0.002
		**Utilities**						
Cooling Water	kg/kgGDP	44.513	33.116	0.852	0.000	0.113	0.169	1.144
Steam		0.000	0.000	0.000	0.000	1.217	0.029	1.834
Power	kWh/kgGDP	0.010	0.224	0.011	0.000	0.010	0.110	0.369
		**Waste**						
Gas Waste	kg/kgGDP	18.281	10.347	3.740	12.774	3.007	2.558	6.016
Solid Waste		0.070	0.058	0.000	0.030	0.000	1.158	0.031
		**Products**						
Power	kWh/kgGDP	0.941	3.289	0.689	0.000	0.569	0.000	0.000
Fertilizer dried	kg/kgGDP	0.0000	0.0000	0.1020	0.0000	0.0830	0.0000	0.0000
Biochar		0.0000	0.0000	0.0000	0.1370	0.0000	0.0000	0.1300
Tannins		0.0000	0.0000	0.0000	0.0000	0.0500	0.0000	0.0000
Oil		0.0000	0.0000	0.0000	0.0000	0.0000	0.0510	0.0510
Polyphenols		0.0000	0.0000	0.0000	0.0000	0.0000	0.0000	0.0380

Table S9: Economic analysis of each process considered

		Combustion	Gasification	Anaerobic Digestion	Pyrolisis	Tannins E	Oil E.	IMPS
				**0.1 kg/s**				
CAPEX	M€	0.849	6.200	1.661	0.395	1.926	4.288	11.077
vOPEX	M€/yr	0.007	0.187	0.005	0.000	0.126	0.010	0.164
fOPEX		0.397	0.959	0.483	0.331	0.815	0.796	1.650
Incomes		0.310	1.020	0.492	0.073	0.706	0.638	4.038
Profit		-0.094	-0.126	0.004	-0.250	-0.236	-0.168	2.224
				**1 kg/s**				
CAPEX	M€	5.140	30.280	4.169	3.368	3.773	17.396	44.930
vOPEX	M€/yr	0.070	1.870	0.070	0.000	0.875	0.103	1.641
fOPEX		0.917	5.040	0.790	0.685	1.605	2.535	7.055
Incomes		3.101	10.200	2.764	0.727	4.907	6.383	40.380
Profit		2.114	3.291	1.904	0.126	2.425	3.746	31.684
				**10 kg/s**				
CAPEX	M€	32.046	160.460	30.369	33.100	32.921	71.574	225.626
vOPEX	M€/yr	0.702	18.702	0.664	0.000	12.521	1.022	16.402
fOPEX		4.859	29.948	4.604	4.224	8.953	10.650	38.448
Incomes		31.012	101.996	91.325	7.273	46.914	63.828	403.810
Profit		25.452	53.337	20.161	3.882	25.440	52.156	348.960

Determination of optimal investment by production capacity

The results of the sensitivity analysis comparing the profitability of each process as a function of its CAPEX are shown in Figure S2.

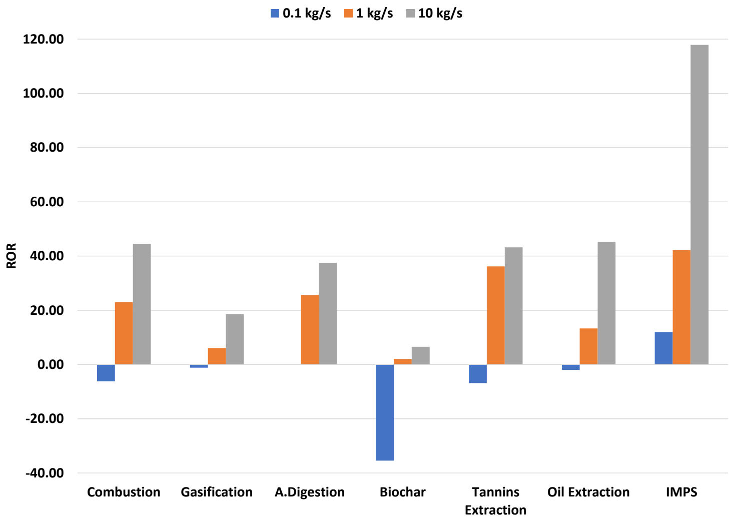

Figure S2: Profitability of each process for each capacity considered

References

(1) Ateş, F.; Büyüktuncer, H.; Yaşar, B.; Işık, A.; Biricik, G.; Koparal, A. S. Comparison of non-catalytic and catalytic fast pyrolysis of pomegranate and grape marcs under vacuum and inert atmospheres. *Fuel* **2019**, *255*, 115788, DOI: 10.1016/j.fuel.2019.115788.

(2) Sinnot, R. *Chemical Engineering Design*, 6th ed.; Elsevier, 2005; Vol. 6; pp 339–359.

(3) León, E.; Martín, M. Optimal production of power in a combined cycle from manure based biogas. *Energy Conversion and Management* **2016**, *114*, 89–99, DOI: 10.1016/j.enconman.2016.02.002.

(4) Sanchez, A.; Martin, M.; Vega, P. Biomass Based Sustainable Ammonia Production: Digestion vs Gasification. *ACS Sustainable Chemistry and Engineering* **2019**, *7*, 9995–10007, DOI: 10.1021/acssuschemeng.9b01158.

(5) Blasi, C. D. Modeling wood gasification in a countercurrent fixed-bed reactor. *AIChE Journal* **2004**, *50*, 2306–2319, DOI: 10.1002/aic.10189.

(6) Taifouris, M.; Martín, M. Multiscale scheme for the optimal use of residues for the production of biogas across Castile and Leon. *Journal of Cleaner Production* **2018**, *185*, 239–251, DOI: 10.1016/J.JCLEPRO.2018.03.018.

(7) Taifouris, M.; Martín, M. Towards energy security by promoting circular economy: A holistic approach. *Applied Energy* **2023**, *333*, 120544, DOI: 10.1016/J.APENERGY.2022.120544.

(8) Converti, A.; Borghi, A. D.; Zilli, M.; Arni, S.; Borghi, M. D. Anaerobic digestion of the vegetable fraction of municipal refuses: Mesophilic versus thermophilic conditions. *Bioprocess Engineering* **1999**, *21*, 371–376, DOI: 10.1007/S004490050689.

(9) Liu, K.; Tang, Y. Q.; Matsui, T.; Morimura, S.; Wu, X. L.; Kida, K. Thermophilic anaerobic co-digestion of garbage, screened swine and dairy cattle manure. *Journal of Bioscience and Bioengineering* **2009**, *107*, 54–60, DOI: 10.1016/J.JBIOSC.2008.09.007.

(10) Bah, H.; Zhang, W.; Wu, S.; Qi, D.; Kizito, S.; Dong, R. Evaluation of batch anaerobic co-digestion of palm pressed fiber and cattle manure under mesophilic conditions. *Waste Management* **2014**, *34*, 1984–1991, DOI: 10.1016/J.WASMAN.2014.07.015.

(11) Rao, M. S.; Singh, S. P. Bioenergy conversion studies of organic fraction of MSW: kinetic studies and gas yield–organic loading relationships for process optimisation. *Bioresource Technology* **2004**, *95*, 173–185, DOI: 10.1016/J.BIORTECH.2004.02.013.

(12) Nielfa, A.; Cano, R.; Vinot, M.; Fernández, E.; Fdz-Polanco, M. Anaerobic digestion modeling of the main components of organic fraction of municipal solid waste. *Process Safety and Environmental Protection* **2015**, *94*, 180–187, DOI: 10.1016/J.PSEP.2015.02.002.

(13) Zhang, W.; Wei, Q.; Wu, S.; Qi, D.; Li, W.; Zuo, Z.; Dong, R. Batch anaerobic co-digestion of pig manure with dewatered sewage sludge under mesophilic conditions. *Applied Energy* **2014**, *128*, 175–183, DOI: 10.1016/J.APENERGY.2014.04.071.

(14) Achkar, J. H. E.; Lendormi, T.; Hobaika, Z.; Salameh, D.; Louka, N.; Maroun, R. G.; Lanoisellé, J. L. Anaerobic digestion of grape pomace: Biochemical characterization of the fractions and methane production in batch and continuous digesters. *Waste Management* **2016**, *50*, 275–282, DOI: 10.1016/j.wasman.2016.02.028.

(15) Almeida, P. V.; Rodrigues, R. P.; Mendes, C. V.; Szelag, R.; Pietrzyk, D.; Klepacz-Smolka, A.; Quina, M. J. Assessment of NIR spectroscopy for predicting biochemical methane potential of agro-residues – A biorefinery approach. *Biomass and Bioenergy* **2021**, *151*, 106169, DOI: 10.1016/j.biombioe.2021.106169.

(16) Javier, H.; Ángel, S. J.; Aida, G.; del Carmen, G. M.; de los Ángeles, M. M. Revalorization of grape marc waste from liqueur wine: biomethanization. *Journal of Chemical Technology & Biotechnology* **2019**, *94*, 1499–1508, DOI: 10.1002/jctb.5909.

(17) Juráček, M.; Vašeková, P.; Massányi, P.; Kováčik, A.; Bíro, D.; Šimko, M.; Gálik, B.; Rolinec, M.; Hanušovský, O.; Kolláthová, R.; Mixtajová, E.; Kalúzová, M. The Effect of Dried Grape Pomace Feeding on Nutrients Digestibility and Serum Biochemical Profile of Wethers. *Agriculture* **2021**, *11*, 1194, DOI: 10.3390/agriculture11121194.

(18) Jin, Q.; O'Keefe, S. F.; Stewart, A. C.; Neilson, A. P.; Kim, Y. T.; Huang, H. Techno-economic analysis of a grape pomace biorefinery: Production of seed oil, polyphenols, and biochar. *Food and Bioproducts Processing* **2021**, *127*, 139–151, DOI: 10.1016/j.fbp.2021.02.002.

(19) Llobera, A.; Cañellas, J. Dietary fibre content and antioxidant activity of Manto Negro red grape (Vitis vinifera): pomace and stem. *Food Chemistry* **2007**, *101*, 659–666, DOI: 10.1016/j.foodchem.2006.02.025.

(20) Wu, L. J.; Kobayashi, T.; Li, Y. Y.; Xu, K. Q. Comparison of single-stage and temperature-phased two-stage anaerobic digestion of oily food waste. *Energy Conversion and Management* **2015**, *106*, 1174–1182, DOI: 10.1016/j.enconman.2015.10.059.

(21) Xu, R.; Ferrante, L.; Briens, C.; Berruti, F. Flash pyrolysis of grape residues into biofuel in a bubbling fluid bed. *Journal of Analytical and Applied Pyrolysis* **2009**, *86*, 58–65, DOI: 10.1016/j.jaap.2009.04.005.

(22) Rodrigues, R. P.; Gando-Ferreira, L. M.; Quina, M. J. Increasing Value of Winery Residues through Integrated Biorefinery Processes: A Review. *Molecules* **2022**, *27*, 4709, DOI: 10.3390/molecules27154709.

(23) Erkac, G.; Yigitarslan, S. Polyphenol Production via Newly Designed System Capable Of Realizing Simultaneous Extraction-Distillation Operations In A Single Column. *IOSR Journal of Applied Chemistry (IOSR-JAC)* **2021**, *14*, 50–63, DOI: 10.9790/5736-1401015063.

(24) Becker, B.; Fricke, B. *FREEZING — Principles*; Elsevier, 2003; pp 2706–2711, DOI: 10.1016/b0-12-227055-x/00521-6, 2706-2711.

(25) Ramos, J. S.; Ferreira, A. F. Techno-economic analysis and life cycle assessment of olive and wine industry co-products valorisation. *Renewable and Sustainable Energy Reviews* **2022**, *155*, 111929, DOI: 10.1016/j.rser.2021.111929.

(26) Sridhar, A.; Kapoor, A.; Kumar, P. S.; Ponnuchamy, M.; Balasubramanian, S.; Prabhakar, S. Conversion of food waste to energy: A focus on sustainability and life cycle assessment. *Fuel* **2021**, *302*, 121069, DOI: 10.1016/j.fuel.2021.121069.

(27) Jin, Q.; O'Keefe, S. F.; Stewart, A. C.; Neilson, A. P.; Kim, Y. T.; Huang, H. Techno-economic analysis of a grape pomace biorefinery: Production of seed oil, polyphenols, and biochar. *Food and Bioproducts Processing* **2021**, *127*, 139–151, DOI: 10.1016/j.fbp.2021.02.002.

(28) ECHEMI, Sodium Sulfite Anhydrous. 2023; https://www.echemi.com/produce/pr2005091011-selling-the-best-quality-manufacture-produce-cost-effective-price-products-96-anhydrous-sodium-sulfite-with-cas-7757-83-7.html, Date accessed: 22/03/2023.

(29) Winnipeg, Emission factors in kg CO2-equivalent per unit. 2022; https://legacy.winnipeg.ca/finance/findata/matmgt/documents/2012/682-2012/682-2012_appendix_h-wstp_south_end_plant_process_selection_report/appendix%207.pdf, Date accessed: 15/11/2022.

(30) Sinnot, R. *Chemical Engineering Design*, 6th ed.; Elsevier, 2005; Vol. 6; pp 339–359, 339-359.

(31) Couper, J. R.; Penney, W. R.; Fair, J. R.; Walas, S. M. *Chemical Process Equipment*, 2nd ed.; Elsevier Inc., 2005; Vol. 1; pp 719–728, DOI: `10.1016/B978-0-7506-7510-9.X5000-1`, 719-728.

(32) Sánchez, A.; Martín, M.; Vega, P. Biomass Based Sustainable Ammonia Production: Digestion vs Gasification. *ACS Sustainable Chemistry and Engineering* **2019**, *7*, 9995–10007, DOI: `10.1021/acssuschemeng.9b01158`.

(33) Almena, A.; Martín, M. Technoeconomic Analysis of the Production of Epichlorohydrin from Glycerol. *Industrial and Engineering Chemistry Research* **2016**, *55*, 3226–3238, DOI: `10.1021/acs.iecr.5b02555`.

(34) Taifouris, M.; Martín, M. Towards energy security by promoting circular economy: A holistic approach. *Applied Energy* **2023**, *333*, 120544, DOI: `10.1016/j.apenergy.2022.120544`.

(35) Ramos, J. S.; Ferreira, A. F. Techno-economic analysis and life cycle assessment of olive and wine industry co-products valorisation. *Renewable and Sustainable Energy Reviews* **2022**, *155*, 111929, DOI: `10.1016/j.rser.2021.111929`.

(36) Jin, Q.; O'Keefe, S. F.; Stewart, A. C.; Neilson, A. P.; Kim, Y. T.; Huang, H. Techno-economic analysis of a grape pomace biorefinery: Production of seed oil, polyphenols, and biochar. *Food and Bioproducts Processing* **2021**, *127*, 139–151, DOI: `10.1016/j.fbp.2021.02.002`.
